# Maternal characteristics and neonatal outcomes in twin pregnancies in Brazil: A nationwide population-based study, 2015–2024

**DOI:** 10.1371/journal.pone.0355195

**Published:** 2026-08-31

**Authors:** Thaísa Bissoli, Thais Rangel Bousquet Carrilho, Nathalia Ferreira Antunes de Almeida, Fernanda Rebelo

**Affiliations:** 1 Graduate Program in Child and Women’s Health, National Institute of Women’s, Children’s and Adolescents’ Health Fernandes Figueira (IFF), Oswaldo Cruz Foundation (Fiocruz), Rio de Janeiro, Brazil; 2 Department of Obstetrics and Gynaecology, Faculty of Medicine, University of British Columbia, Vancouver, Canada; 3 Department of Applied Nutrition, Federal University of the State of Rio de Janeiro (UNIRIO), Rio de Janeiro, Brazil; 4 Graduate Program in Food and Nutrition Security (PPGSAN), Federal University of the State of Rio de Janeiro (UNIRIO), Rio de Janeiro, Brazil; 5 Clinical Research Unit, National Institute of Women’s, Children’s and Adolescents’ Health Fernandes Figueira (IFF), Oswaldo Cruz Foundation (Fiocruz), Rio de Janeiro, Brazil; Federal University of Santa Maria: Universidade Federal de Santa Maria, BRAZIL

## Abstract

**Objective:**

To describe maternal characteristics and neonatal outcomes of twin pregnancies in Brazil, nationwide and across macro-regions, between 2015 and 2024.

**Methods:**

This descriptive study used data from the Brazilian Live Birth Information System (SINASC). All live births from twin pregnancies with gestational age ≥ 24 weeks, maternal age 19–49 years, and complete information on birth weight and gestational age were included. Deterministic record linkage was applied to identify complete twin pairs. Maternal characteristics included age, education, prenatal care utilization, marital status, race/skin color, region of residence, and mode of delivery. Neonatal outcomes included low birth weight (<2,500 g), preterm birth (<37 weeks), and birth weight discordance (≥ 20%). Frequencies and corresponding 95% confidence intervals were estimated by year and macro-region.

**Results:**

A total of 437,728 live births (218,864 complete twin pairs) were included. Most mothers were aged 25–34 years (53.1%), had 8–11 years of schooling (56.0%), attended seven or more prenatal visits (75.3%), and had a cesarean delivery (87.5%). Higher concentrations of twin births were observed in the Southeast and Northeast regions. Approximately 60% of newborns had low birth weight and were born preterm, and these outcomes were more frequent in the South and Southeast regions. Birth weight discordance ≥ 20% occurred in approximately 16% of twin pairs and showed limited regional variation.

**Conclusion:**

Twin pregnancies in Brazil present a substantial burden of adverse neonatal outcomes and marked regional inequalities. Strengthening specialized prenatal and perinatal care and adopting twin-specific clinical and epidemiological criteria are essential to improve risk assessment and optimize care for this high-risk population.

## Introduction

In recent decades, a significant increase in the rate of twin births has been observed worldwide, rising from 9.1 to 12.0 per 1,000 births between 1980–1985 and 2010–2015 [1]. This increase has been mainly attributed to the expanded use of assisted reproductive technologies, delayed childbearing, and higher maternal body mass index (BMI) [2–4]. In Brazil, the proportion of twin births increased from 1.87% in 2012 to 2.19% in 2022 [5].

Twin pregnancies are consistently associated with a higher risk of adverse neonatal outcomes, including low birth weight, preterm birth, intrauterine growth restriction, and neonatal morbidity and mortality [6,7]. In addition, maternal risks are increased, with higher incidences of hypertensive disorders, hemorrhage, and cesarean delivery [8,9]. These risks result in greater utilization of health services, including longer hospital stays, increased obstetric interventions, and higher demand for neonatal intensive care resources [10]. Given the clinical and epidemiological complexity of twin gestations, it is essential to characterize maternal profiles, monitor neonatal outcomes, and understand patterns of care in this population.

In Brazil, the Live Birth Information System (SINASC, from the Portuguese acronym) is a strategic tool for monitoring birth patterns and perinatal indicators. The SINASC provides standardized nationwide data on maternal, gestational, and neonatal characteristics through the Live Birth Certificate [11,12], enabling the identification of epidemiological trends and regional inequalities, supporting health system planning and policy development.

Although studies conducted in Brazil have described perinatal outcomes in twin pregnancies, most available evidence derives from hospital-based cohorts or regional studies [13]. Nationwide population-based analyses covering recent years remain limited. Thus, this study aimed to describe maternal characteristics and neonatal outcomes of twin pregnancies in Brazil, at the national level and across macro-regions, between 2015 and 2024, using data from the SINASC. By analyzing a decade of population-based records, this study provides an updated overview of twin pregnancies in a large middle-income country with substantial regional inequalities and generates evidence that can inform maternal and neonatal health policies in other low- and middle-income settings facing similar challenges in perinatal care.

## Methods

### Study design, population, and eligibility criteria

This descriptive, cross-sectional study was based on publicly available secondary data from the Live Birth Information System (SINASC), provided by the Department of Informatics of the Brazilian Unified Health System (DATASUS). SINASC is a nationwide information system established by the Brazilian Ministry of Health to systematically collect data on live births across the country using standardized birth certificates completed at the time of delivery. The analysis included anonymized individual-level records of live births occurring between 2015 and 2024, covering all regions of Brazil. These data are publicly accessible through the DATASUS platform (https://datasus.saude.gov.br/), allowing unrestricted access for research purposes. Data were accessed in December 2025. The dataset was fully anonymized, and no identifiable information was available to the authors during or after data collection.

All records of live births from twin pregnancies during the study period were eligible for inclusion. Triplet or higher-order multiple pregnancies were excluded. Records with missing or inconsistent information in key variables, including gestational age, sex, and birth weight, were also excluded. Gestational age values below 168 days (24 completed weeks) or above 301 days (43 completed weeks) were excluded according to prespecified eligibility criteria. This range is consistent with procedures adopted by the World Health Organization [14], and with previous registry-based studies of twin pregnancies [15]. Extreme birth weight values were identified using sex and gestational age-specific z-scores, and records exceeding ±5 standard deviations were considered biologically implausible and excluded [16]. Similar criteria have been applied in studies involving twin populations [17].

### Identification of twin pairs

The identification of twin pairs was performed using a rule-based deterministic record linkage strategy based on the exact agreement of multiple maternal and birth-related variables with high discriminative power, as recommended for large administrative databases in the absence of unique identifiers [18,19]. Variables used for linkage included maternal age, marital status, maternal education, number of prenatal visits, date of birth, municipality of birth, state of maternal birthplace, gestational age, maternal race/skin color, and maternal occupation. Records sharing the same combination of these variables were classified as belonging to the same pregnancy [20]. As publicly available SINASC microdata do not include a unique maternal or pregnancy identifier, the linkage relied on the combination of these variables to infer records belonging to the same pregnancy. Only complete twin pairs, defined as exactly two live birth records linked to the same pregnancy, were retained for analysis. To further ensure linkage consistency and minimize spurious matches, temporal proximity between births was assessed using the recorded date and time of delivery. Pairs with implausible interbirth intervals greater than 24 hours were excluded from the study population.

### Main variables

Population characterization included maternal characteristics (age, education, marital status, number of prenatal visits, race/skin color, and macro-region of residence) and neonatal descriptors (sex, mode of delivery, and race/skin color). Maternal age was categorized into 19–24, 25–34, and ≥35 years. Maternal education was grouped into none, 1–7, 8–11, and ≥12 schooling years. The number of prenatal visits was categorized as none, 1–6, and ≥7 visits. Marital status was classified as living with or without a partner. Maternal race/skin color and region of residence were classified according to the categories available in the SINASC (white, parda [mixed race], black, asian, and indigenous).

Birth weight was obtained from SINASC records and corresponds to the newborn's weight recorded at delivery in the Declaration of Live Birth. Low birth weight was defined as birth weight <2,500 g [21]. Preterm birth was defined as gestational age at delivery <37 weeks [22]. Gestational age, expressed in completed weeks, was obtained from SINASC records and reflects the best estimate available at birth registration. According to the instructions for completing the Declaration of Live Birth, when the date of the last menstrual period is unavailable, gestational age may be estimated using other methods, including ultrasonography or clinical examination. However, the specific method used to determine gestational age for each individual birth is not available in the public SINASC database [12].

Twin birth weight discordance was calculated for each pair using the formula: [(birth weight of the larger twin − birth weight of the smaller twin) / birth weight of the larger twin] × 100. The discordance was then categorized as < 20% and ≥ 20%, according to clinically relevant thresholds associated with increased risk of adverse neonatal outcomes [23,24].

### Statistical analysis

Categorical variables were presented as absolute and relative frequencies with corresponding 95% confidence intervals (95% CI). Comparisons between groups and across study years were based on the magnitude of observed differences and their confidence intervals. Because twins share the same pregnancy and, consequently, maternal characteristics, the potential lack of independence between newborns was considered during the analytical strategy. For outcomes defined at the pregnancy level, such as preterm birth and birth weight discordance, each twin pair represents a single biological context, thus comparisons using proportions and confidence intervals are appropriate for the purposes of this study. Temporal variation was assessed through annual frequencies and graphical presentation of the main indicators to identify clinically-relevant changes over time. All analyses were performed using R software, version 4.5.1.

### Ethics statement

This study used publicly available, anonymized secondary data. According to Brazilian regulations, studies using public, de-identified databases do not require approval by a Research Ethics Committee.

## Results

**Fig 1:**
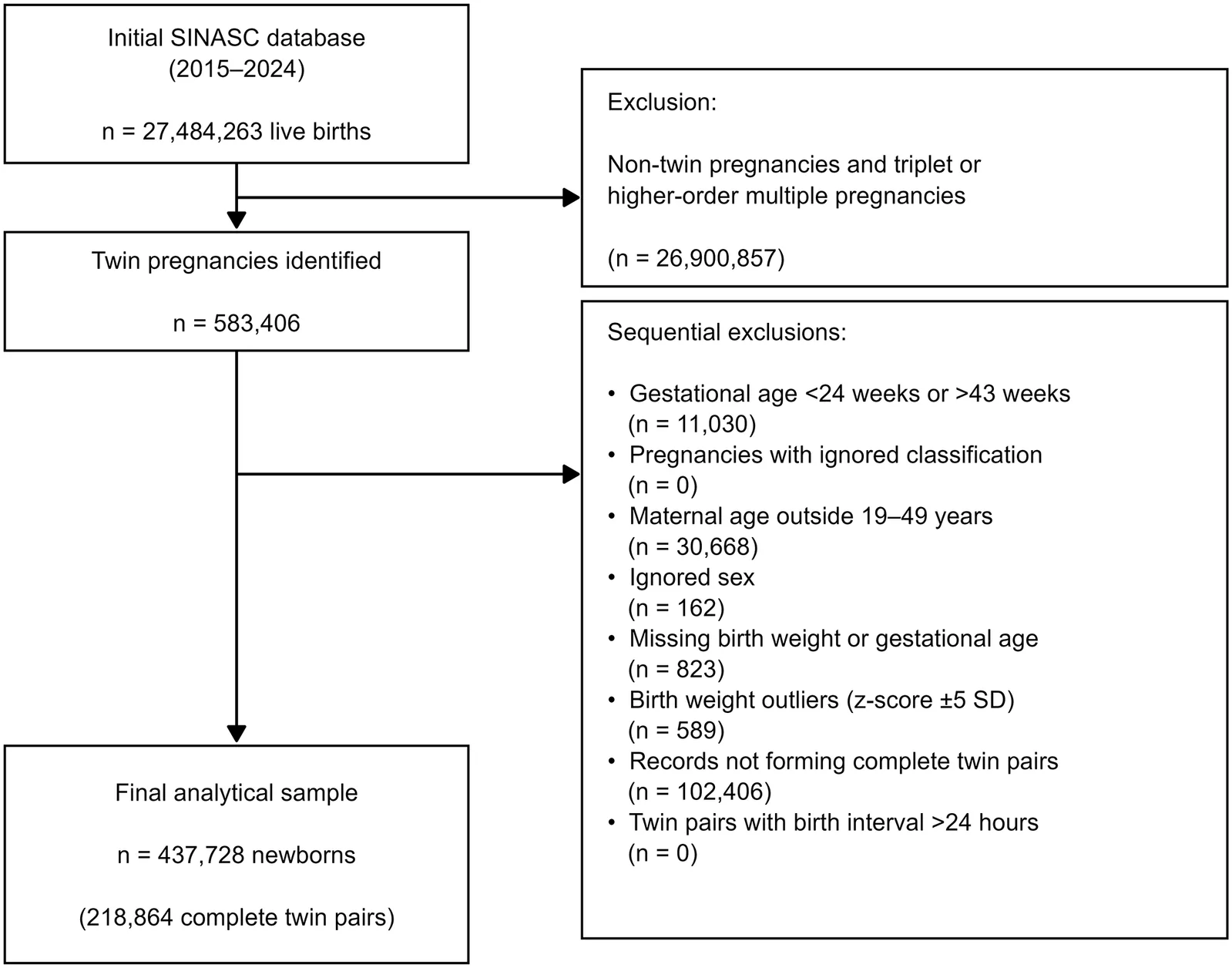
Flowchart of participant selection in the database and successive exclusions.

The SINASC database comprised 27,484,263 live births registered in Brazil between 2015 and 2024. After selecting records from twin pregnancies, 583,406 live births were identified. After all eligibility criteria and exclusions were applied, the final study population comprised 437,728 live births, corresponding to 218,864 complete twin pairs (Fig 1).

**Fig 2:**
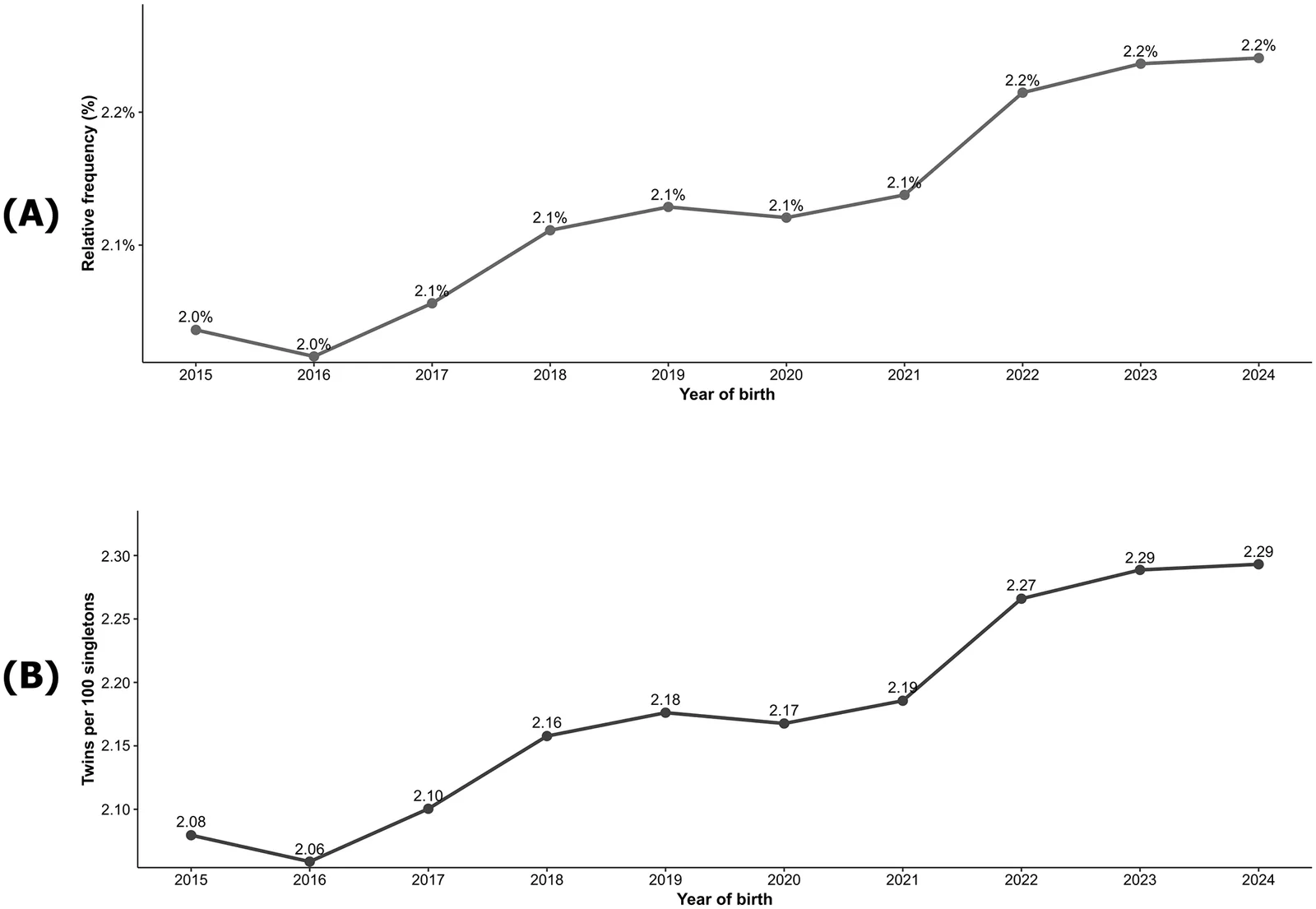
Temporal trends in twin births in Brazil, 2015–2024. (A) Proportion (%) of twin births among all live births by year of birth. (B) Ratio of twin births per 100 singleton births by year of birth.

Between 2015 and 2024, the proportion of twin births in Brazil increased from 2.0% to 2.2%, corresponding to an absolute increase of 0.2 percentage points and a relative increase of 10%. Over the same period, the ratio of twin to singleton births rose from 2.08 to 2.29 per 100 singleton births, representing a relative increase of approximately 10% (Fig 2).

### Maternal characteristics and neonatal descriptors

**Fig 3:**
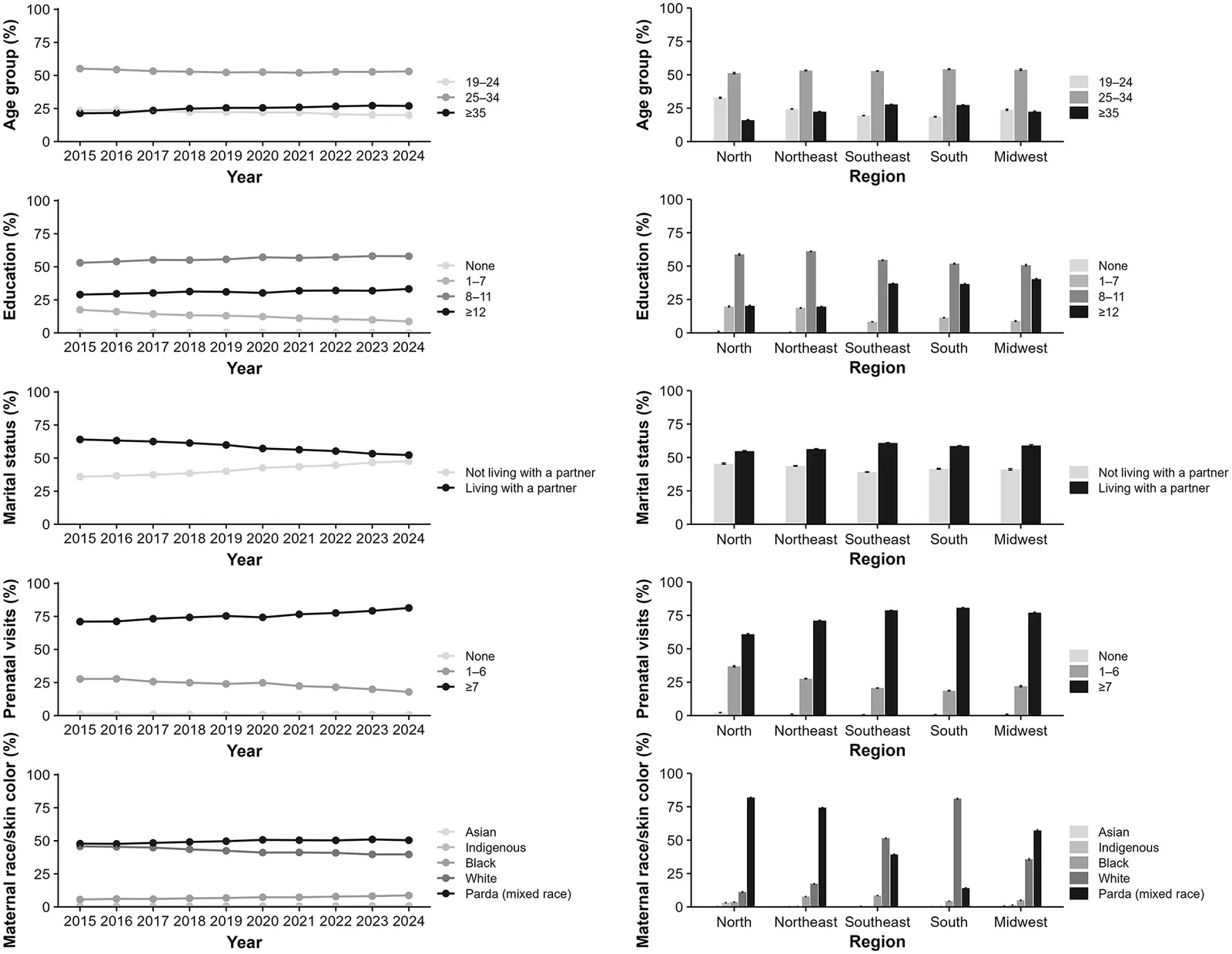
Frequency (%) of maternal sociodemographic characteristics among twin pregnancies in Brazil by year of birth (2015–2024) and macro-region.

Most twin pregnancies occurred among women aged 25–34 years (53.1%, 95% CI 52.9–53.3). Over the study period, there was a progressive increase in the proportion of women aged ≥35 years, accompanied by a decline in younger age groups. Most mothers had 8–11 schooling years (56.0%, 95% CI 55.8–56.2). Over time, the proportion of women with ≥12 years of education increased, accompanied by a decline in the proportions with no formal education and with 1–7 years of schooling. Most women reported living with a partner (58.6%, 95% CI 58.4–58.8), although this proportion showed a gradual decrease across the study period. In terms of maternal race/skin color, most mothers (49.4%) were parda (mixed race), with marked regional differences. Higher proportions of white women were observed in the South and Southeast, whereas parda women were more prevalent in the North and Northeast. Prenatal care coverage was high, with 75.3% (95% CI 75.1–75.5) of women attending seven or more prenatal visits, showing a consistent upward trend over time (Fig 3; S1 Table).

**Fig 4:**
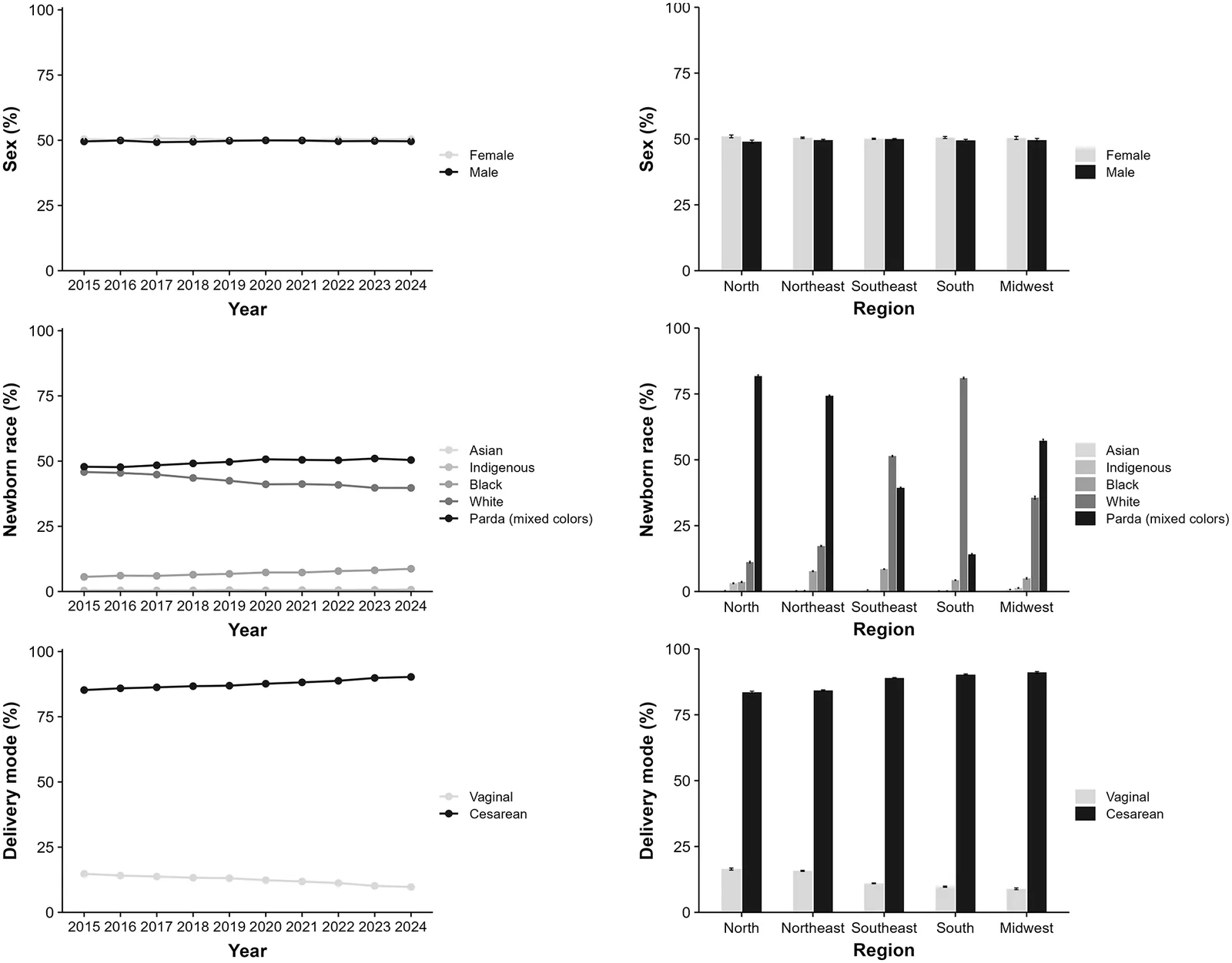
Neonatal characteristics and mode of delivery by year of birth and Brazilian macro-region, 2015–2024.

Among newborns, the distribution of sex remained stable throughout the study period, with similar proportions of female (50.3%) and male (49.7%) infants. Cesarean delivery was the predominant mode of birth, accounting for 87.5% (95% CI 87.4–87.6) of all deliveries, and increased over time, exceeding 90% in recent years. Cesarean rates were consistently high across all macro-regions, with the highest proportions observed in the South, Southeast, and Central-West, and slightly lower, though still elevated, rates in the North and Northeast (Fig 4).

### Neonatal outcomes

**Fig 5:**
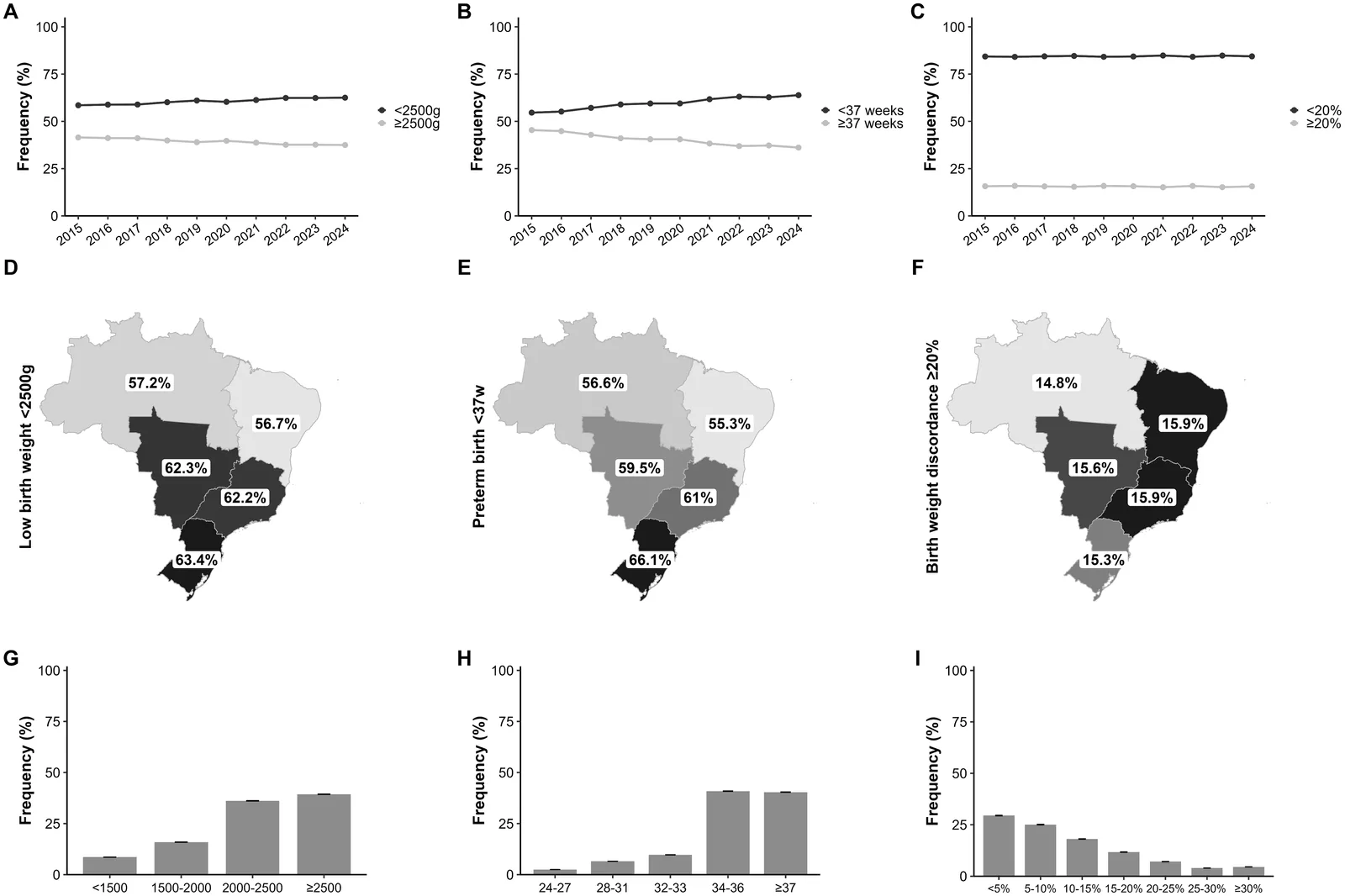
Unified neonatal outcomes by year and macro-region, 2015–2024. (A) Low birth weight (<2500 g). (B) Preterm birth (<37 weeks). (C) Birth weight discordance (<20% and ≥20%). (D) Low birth weight (<2500 g) by macro-region. (E) Preterm birth (<37 weeks) by macro-region. (F) Birth weight discordance (≥20%) by macro-region. (G) Birth weight categories. (H) Gestational age categories. (I) Birth weight discordance categories.

Low birth weight (<2,500 g) and preterm birth (<37 weeks) were frequent among twin newborns throughout the study period. Overall, both outcomes affected approximately 60% of live births. Temporal analyses indicated a gradual increase in the frequency of low birth weight and preterm birth between 2015 and 2024 (Figs 5A and 5B). Regionally, higher frequencies of preterm birth were observed in the South and Southeast, followed by the Central-West, whereas lower frequencies were found in the North and Northeast (Fig 5E). Similar regional distribution was observed for low birth weight (Fig 5D). These regional patterns remained consistent over time.

Most twin pairs (84.3%) exhibited birth weight discordance < 20%. Discordance ≥ 20% was observed in approximately 15–16% of twin pairs and did not show a clear temporal trend over the study period (Fig 5C). The frequency of birth weight discordance ≥ 20% was similar across Brazilian macro-regions, ranging from 14.8% in the North to 15.9% in the Northeast and Southeast (Fig 5F).

### Distribution of neonatal outcomes according to maternal and pregnancy characteristics

Neonatal outcomes varied according to maternal and pregnancy characteristics (Table 1) and showed consistent patterns over time and across regions (Fig 5). Low birth weight occurred in 63.2% (95% CI 62.9–63.5) of newborns born to mothers aged 19–24 years, 59.4% (95% CI 59.2–59.6) among those aged 25–34 years, and 61.0% (95% CI 60.7–61.3) among those aged ≥35 years. Preterm birth followed a similar pattern, with frequencies of 59.4% (95% CI 59.1–59.7), 58.8% (95% CI 58.6–59.0), and 61.6% (95% CI 61.3–61.9), respectively. Confidence intervals overlapped across age groups. Birth weight discordance ≥ 20% showed minimal variation by maternal age, with overlapping confidence intervals.

**Table 1:** Distribution of maternal and neonatal characteristics according to neonatal outcomes in twin pregnancies in Brazil: low birth weight, preterm birth, and intrapair birth weight discordance. SINASC, 2015–2024.

Maternal and newborn characteristics		Low Birth Weight (<2500 g)		Preterm (<37 weeks)		Weight Discordance (≥ 20%)	
		No	Yes	No	Yes	No	Yes
Maternal age group							
	19–24	35458 (20.6% [20.4–20.8%])	60898 (22.9% [22.8–23.1%])	39096 (22.1% [21.9–22.3%])	57260 (21.9% [21.8–22.1%])	82412 (22.3% [22.2–22.4%])	13944 (20.4% [20.1–20.7%])
	25–34	94362 (54.7% [54.5–55%])	138058 (52% [51.8–52.2%])	95846 (54.2% [54–54.5%])	136574 (52.3% [52.1–52.5%])	196930 (53.3% [53.1–53.5%])	35490 (52% [51.7–52.4%])
	≥ 35	42532 (24.7% [24.5–24.9%])	66420 (25% [24.9–25.2%])	41820 (23.7% [23.5–23.9%])	67132 (25.7% [25.6–25.9%])	90176 (24.4% [24.3–24.5%])	18776 (27.5% [27.2–27.9%])
Number of prenatal visits							
	None	1200 (0.7% [0.7–0.7%])	3094 (1.2% [1.1–1.2%])	1444 (0.8% [0.8–0.9%])	2850 (1.1% [1.1–1.1%])	3562 (1% [0.9–1%])	732 (1.1% [1–1.2%])
	1-6	29262 (17% [16.9–17.2%])	74018 (28% [27.9–28.2%])	30048 (17.1% [16.9–17.2%])	73232 (28.2% [28–28.4%])	85694 (23.3% [23.2–23.4%])	17586 (25.9% [25.6–26.3%])
	7 or more	141182 (82.3% [82.1–82.4%])	186846 (70.8% [70.6–71%])	144590 (82.1% [81.9–82.3%])	183438 (70.7% [70.5–70.9%])	278508 (75.7% [75.6–75.9%])	49520 (73% [72.7–73.3%])
Maternal education (schooling years)							
	None	282 (0.3% [0.3–0.4%])	438 (0.3% [0.3–0.4%])	365 (0.4% [0.4–0.5%])	355 (0.3% [0.2–0.3%])	577 (0.3% [0.3–0.3%])	143 (0.4% [0.4–0.5%])
	1–7	11563 (13.1% [12.8–13.3%])	16118 (12.5% [12.3–12.6%])	12660 (14.4% [14.2–14.6%])	15021 (11.6% [11.4–11.7%])	23234 (12.6% [12.5–12.8%])	4447 (13.1% [12.7–13.5%])
	8-11	50038 (56.5% [56.2–56.9%])	72007 (55.6% [55.4–55.9%])	50434 (57.4% [57–57.7%])	71611 (55.1% [54.8–55.3%])	103291 (56.1% [55.9–56.4%])	18754 (55.2% [54.7–55.7%])
	≥12	26641 (30.1% [29.8–30.4%])	40872 (31.6% [31.3–31.8%])	24472 (27.8% [27.5–28.1%])	43041 (33.1% [32.8–33.4%])	56885 (30.9% [30.7–31.1%])	10628 (31.3% [30.8–31.8%])
Marital status							
	Living with a partner	102165 (59.4% [59.2–59.6%])	153947 (58.1% [57.9–58.3%])	102474 (58.1% [57.9–58.3%])	153638 (59% [58.8–59.2%])	216202 (58.6% [58.5–58.8%])	39910 (58.6% [58.2–59%])
	Not living with a partner	69786 (40.6% [40.4–40.8%])	110908 (41.9% [41.7–42.1%])	73876 (41.9% [41.7–42.1%])	106818 (41% [40.8–41.2%])	152512 (41.4% [41.2–41.5%])	28182 (41.4% [41–41.8%])
Maternal race/ skin color							
	White	68064 (40.5% [40.3–40.7%])	113122 (43.7% [43.5–43.9%])	66054 (38.4% [38.1–38.6%])	115132 (45.2% [45–45.4%])	153084 (42.5% [42.3–42.6%])	28102 (42.2% [41.9–42.6%])
	‘Parda’ (mixed race)	86229 (51.3% [51.1–51.6%])	124627 (48.1% [47.9–48.3%])	91092 (52.9% [52.7–53.2%])	119764 (47% [46.8–47.2%])	177952 (49.4% [49.2–49.5%])	32904 (49.5% [49.1–49.8%])
	Black	12035 (7.2% [7–7.3%])	18717 (7.2% [7.1–7.3%])	13070 (7.6% [7.5–7.7%])	17682 (6.9% [6.8–7%])	25874 (7.2% [7.1–7.3%])	4878 (7.3% [7.1–7.5%])
	Indigenous	979 (0.6% [0.5–0.6%])	1205 (0.5% [0.4–0.5%])	1134 (0.7% [0.6–0.7%])	1050 (0.4% [0.4–0.4%])	1844 (0.5% [0.5–0.5%])	340 (0.5% [0.5–0.6%])
	Asian	720 (0.4% [0.4–0.5%])	1260 (0.5% [0.5–0.5%])	758 (0.4% [0.4–0.5%])	1222 (0.5% [0.5–0.5%])	1672 (0.5% [0.4–0.5%])	308 (0.5% [0.4–0.5%])
Macro-Region							
	Central-West	10804 (6.4% [6.3–6.5%])	17498 (6.7% [6.6–6.8%])	11202 (6.4% [6.3–6.6%])	17100 (6.6% [6.5–6.7%])	23944 (6.6% [6.5–6.7%])	4358 (6.5% [6.3–6.7%])
	Northeast	52157 (30.7% [30.5–31%])	70017 (26.7% [26.6–26.9%])	53880 (31% [30.8–31.2%])	68294 (26.5% [26.3–26.7%])	102926 (28.3% [28.1–28.4%])	19248 (28.6% [28.3–28.9%])
	North	14361 (8.5% [8.3–8.6%])	19333 (7.4% [7.3–7.5%])	15262 (8.8% [8.6–8.9%])	18432 (7.2% [7.1–7.3%])	28558 (7.8% [7.8–7.9%])	5136 (7.6% [7.4–7.8%])
	Southeast	67705 (39.9% [39.7–40.1%])	112951 (43.1% [42.9–43.3%])	69930 (40.2% [40–40.5%])	110726 (43% [42.8–43.2%])	152476 (41.9% [41.7–42%])	28180 (41.9% [41.5–42.2%])
	South	24624 (14.5% [14.3–14.7%])	42092 (16.1% [15.9–16.2%])	23528 (13.5% [13.4–13.7%])	43188 (16.8% [16.6–16.9%])	56344 (15.5% [15.4–15.6%])	10372 (15.4% [15.1–15.7%])
Mode of delivery							
	Cesarean	156987 (91.1% [91–91.2%])	226138 (85.2% [85.1–85.4%])	159254 (90.1% [90–90.3%])	223871 (85.8% [85.7–85.9%])	322442 (87.3% [87.2–87.4%])	60683 (89% [88.8–89.2%])
	Vaginal	15327 (8.9% [8.8–9%])	39191 (14.8% [14.6–14.9%])	17463 (9.9% [9.7–10%])	37055 (14.2% [14.1–14.3%])	47011 (12.7% [12.6–12.8%])	7507 (11% [10.8–11.2%])
Infant sex							
	Female	78390 (45.5% [45.2–45.7%])	141899 (53.5% [53.3–53.7%])	89781 (50.8% [50.6–51%])	130508 (50% [49.8–50.2%])	185924 (50.3% [50.2–50.5%])	34365 (50.4% [50–50.8%])
	Male	93962 (54.5% [54.3–54.8%])	123477 (46.5% [46.3–46.7%])	86981 (49.2% [49–49.4%])	130458 (50% [49.8–50.2%])	183594 (49.7% [49.5–49.8%])	33845 (49.6% [49.2–50%])
Infant race/skin color							
	Black	12050 (7.1% [7–7.3%])	18729 (7.2% [7.1–7.3%])	13086 (7.6% [7.4–7.7%])	17693 (6.9% [6.8–7%])	25898 (7.2% [7.1–7.2%])	4881 (7.3% [7.1–7.5%])
	‘Parda’ (mixed race)	86737 (51.4% [51.2–51.7%])	125232 (48.2% [48–48.4%])	91821 (53.1% [52.8–53.3%])	120148 (47.1% [46.9–47.2%])	178894 (49.5% [49.3–49.6%])	33075 (49.5% [49.2–49.9%])
	White	68183 (40.4% [40.2–40.7%])	113285 (43.6% [43.4–43.8%])	66223 (38.3% [38–38.5%])	115245 (45.1% [44.9–45.3%])	153324 (42.4% [42.2–42.6%])	28144 (42.2% [41.8–42.5%])
	Indigenous	987 (0.6% [0.5–0.6%])	1215 (0.5% [0.4–0.5%])	1148 (0.7% [0.6–0.7%])	1054 (0.4% [0.4–0.4%])	1856 (0.5% [0.5–0.5%])	346 (0.5% [0.5–0.6%])
	Asian	720 (0.4% [0.4–0.5%])	1260 (0.5% [0.5–0.5%])	758 (0.4% [0.4–0.5%])	1222 (0.5% [0.5–0.5%])	1672 (0.5% [0.4–0.5%])	308 (0.5% [0.4–0.5%])

With respect to maternal education, most low birth weight births occurred among mothers with 8–11 years of schooling (55.6%; 95% CI 55.4–55.9), followed by those with ≥12 years (31.6%; 95% CI 31.3–31.8) and 1–7 years of schooling (12.5%; 95% CI 12.3–12.6). A similar distribution was observed for preterm birth, with corresponding proportions of 55.1% (95% CI 54.8–55.3), 33.1% (95% CI 32.8–33.4), and 11.6% (95% CI 11.4–11.7), respectively. Birth weight discordance ≥ 20% followed the same pattern, with most mothers having 8–11 years of schooling (55.2%; 95% CI 54.7–55.7), followed by ≥12 years (31.3%; 95% CI 30.8–31.8) and 1–7 years (13.1%; 95% CI 12.7–13.5).

The frequencies of neonatal outcomes varied most markedly according to the number of prenatal care visits. Women who attended seven or more prenatal visits had lower frequencies of low birth weight (57.0%; 95% CI 56.8–57.2) and preterm birth (55.9%; 95% CI 55.7–56.1) compared with those with 1–6 visits (low birth weight: 71.7%; 95% CI 71.3–72.1; preterm birth: 70.9%; 95% CI 70.4–71.3) and those with no prenatal visits (low birth weight: 72.0%; 95% CI 70.6–73.4; preterm birth: 66.4%; 95% CI 64.9–67.9). In these comparisons, confidence intervals did not overlap. For birth weight discordance ≥ 20%, frequencies were slightly lower among women with ≥7 visits (15.1%; 95% CI 15.0–15.3) compared with other categories.

No relevant differences were observed in the frequencies of low birth weight, preterm birth, or birth weight discordance ≥ 20% according to maternal marital status or maternal race/skin color. Newborns delivered vaginally had higher frequencies of low birth weight and preterm birth compared with those delivered by cesarean section, with nonoverlapping confidence intervals. In contrast, birth weight discordance ≥20% showed minimal variation according to mode of delivery, with overlapping confidence intervals.

## Discussion

### Main findings

This study provides one of the largest population-based descriptions of twin pregnancies in Brazil, analyzing more than 218,000 twin pairs over a ten-year period using nationwide administrative data. The findings show a high perinatal burden in twin pregnancies, with preterm birth and low birth weight affecting approximately 60% of live births. In addition, important regional differences were observed, with higher frequencies of these outcomes in the South and Southeast regions. Cesarean delivery was the predominant mode of birth, accounting for a very high proportion of twin deliveries and increasing over time. Birth weight discordance ≥ 20% affected approximately 15% of twin pairs and showed limited regional variation.

**Comparison with the literature**The high prevalence of preterm birth and low birth weight observed in this study is consistent with previous evidence showing that multiple pregnancies are associated with a substantially higher risk of adverse outcomes compared with singleton pregnancies [6–9]. The regional distribution of these outcomes in Brazil, with higher levels in more developed regions, should be interpreted in light of previous national evidence showing a greater concentration of assisted reproductive technologies [25], better access to prenatal and obstetric care [26–28], and more intervention-intensive obstetric care practices [29]. These patterns may also reflect broader regional differences in healthcare infrastructure and service organization. Nationwide studies have documented important inequalities in access to obstetric care across Brazil, including disparities in the availability of intensive care units and specialized maternity services, geographic barriers to childbirth care, and limitations in referral and counter-referral systems within the Brazilian Unified Health System [26,27]. Such structural differences may influence access to timely and appropriate care for high-risk pregnancies, including twin gestations, and should be considered when interpreting regional variations in neonatal outcomes.

In addition, an important caveat when interpreting the rates of preterm birth observed among twins in this study is the fact that the SINASC does not include data to differentiate between spontaneous and medically indicated preterm births. In the context of twin pregnancies, this distinction is especially important because many births occurring before 37 weeks may occur due to medical indication, and not necessarily because of spontaneous onset of labor [30,31].

In our study, women aged ≥ 35 years represented a substantial proportion of mothers of twin pregnancies. Previous studies have shown that delayed childbearing contributes to the increasing frequency of twin pregnancies worldwide [1]. This pattern may reflect both biological and technological mechanisms. The risk of spontaneous dizygotic twinning increases with maternal age, possibly reflecting higher concentrations of follicle-stimulating hormone (FSH) and the resulting increased likelihood of multiple ovulation [32], while older women are also more likely to conceive using assisted reproductive technologies, which further contribute to twinning rates [4]. In addition, maternal age and plurality may jointly contribute to higher frequencies of maternal complications and adverse perinatal outcomes among twin pregnancies [9]. Although our descriptive design does not allow assessment of the independent contribution of maternal age to neonatal outcomes, these findings should be interpreted within the broader demographic and reproductive transitions observed in Brazil and globally.

Cesarean delivery was the predominant mode of birth, accounting for more than 87% of deliveries. This finding is consistent with previous reports showing high cesarean rates in twin pregnancies both in Brazil and internationally [6–8,13,30]. The persistently high frequency observed across all regions is also consistent with the obstetric context of Brazil, a country that has historically reported among the highest cesarean rates worldwide [26,29]. However, its interpretation requires caution because it is not possible to determine whether these cesarean deliveries were clinically indicated or potentially avoidable.

Birth weight discordance showed a prevalence consistent with national and international studies [13,24]. However, the absence of information on chorionicity limits more detailed mechanistic interpretations for this outcome. The observed differences in neonatal outcomes according to maternal education and prenatal care utilization should also be interpreted cautiously. Higher educational attainment and a greater number of prenatal visits may reflect differences in healthcare access, referral to specialized services, intensity of surveillance, and underlying maternal and pregnancy characteristics rather than direct effects on neonatal outcomes [26,28].

**Strengths and limitations**This study has several important strengths, including the use of a large nationwide population dataset over a ten-year period, ensuring broad representativeness, and the evaluation of maternal characteristics and neonatal outcomes across all Brazilian macro-regions. The application of a deterministic record linkage strategy enabled the identification of complete twin pairs in publicly available microdata, representing a robust approach for large-scale population analyses and an important methodological contribution to epidemiological studies using the SINASC [19]. Although deterministic linkage may underidentify some true twin pairs, careful variable selection and temporal consistency checks were applied to improve reliability [18,19]. In addition, the simultaneous evaluation of preterm birth, low birth weight, and birth weight discordance provides an integrated overview of key neonatal outcomes in twin pregnancies.

Despite these strengths, some limitations should be considered. The use of administrative data may be subject to measurement errors, incomplete records, or misclassification of variables, and although several data-cleaning procedures were implemented, including the exclusion of twin pairs with biologically implausible gestational ages, residual inaccuracies cannot be completely ruled out. Interpretation of maternal race/skin color should be undertaken with caution due to known limitations in data quality [33,34]. Furthermore, the database lacks important clinical variables, including chorionicity, use of assisted reproductive technologies, detailed obstetric indications for delivery, maternal nutritional status during pregnancy, information on maternal morbidities such as gestational diabetes and hypertensive disorders of pregnancy, and other relevant maternal or fetal conditions that could influence neonatal outcomes.

## Conclusions

The findings of this study have direct implications for the planning and organization of maternal and child healthcare services in Brazil. The high frequency of preterm birth and low birth weight in twin pregnancies reinforces the need to strengthen access to specialized prenatal care, improve referral and counter-referral pathways for high-risk pregnancies, and expand neonatal intensive care capacity. These efforts are particularly relevant in regions with a higher burden of preterm birth and low birth weight, where the demand for specialized obstetric and neonatal services may be greater. At the same time, regional planning should also address inequalities in healthcare infrastructure and access to ensure equitable care for twin pregnancies across the country.

Beyond its implications for Brazil, this study also provides relevant evidence for countries with similar demographic and healthcare characteristics. While most available data on twin pregnancies derive from high-income settings, less is known about patterns of maternal characteristics, healthcare organization, and perinatal outcomes in low- and middle-income countries [35,36]. The findings observed here reflect structural and healthcare inequalities that are common in many such settings. Thus, these results may inform the organization of perinatal care in comparable contexts, especially regarding access to specialized services, management of high-risk pregnancies, and allocation of neonatal intensive care resources.

## Supporting information

S1 Table Distribution of maternal and neonatal characteristics among twin pregnancies in Brazil, 2015–2024. (DOCX)
