# Supplementary material for: Maternal characteristics and neonatal outcomes in twin pregnancies in Brazil: A nationwide population-based study, 2015–2024
Source: PLoS One. 2026 Aug 31;21(8):e0355195. doi: 10.1371/journal.pone.0355195 (PMC13529000; doi:10.1371/journal.pone.0355195)
Supplement: Supplementary file 1 [file pone.0355195.s001.docx]

# 1. Table 1. Distribution of maternal and neonatal characteristics according to neonatal outcomes in twin pregnancies in Brazil: low birth weight, preterm birth, and intrapair birth weight discordance. SINASC, 2015–2024.

Values are n (% [95% CI]) using the Wilson method. Gestational age was restricted to 24–43 weeks.

|  | | No | Yes | No | Yes | No | Yes |
| --- | --- | --- | --- | --- | --- | --- | --- |
| Variable | Category | Low Birth Weight (<2500 g) | | Preterm (<37 weeks) | | Weight Discordance (≥20%) | |
| Variable | Category | LBW_No | LBW_Yes | PRE_No | PRE_Yes | DIS_No | DIS_Yes |
| Maternal age (years) | 19–24 | 18194 (20.5% [20.2–20.7%]) | 29984 (23.1% [22.9–23.3%]) | 19548 (22.1% [21.8–22.4%]) | 28630 (21.9% [21.7–22.2%]) | 41206 (22.3% [22.1–22.5%]) | 6972 (20.4% [20–20.9%]) |
|  | 25–34 | 48686 (54.7% [54.4–55.1%]) | 67524 (52% [51.7–52.2%]) | 47923 (54.2% [53.9–54.6%]) | 68287 (52.3% [52.1–52.6%]) | 98465 (53.3% [53.1–53.5%]) | 17745 (52% [51.5–52.6%]) |
|  | ≥35 | 22064 (24.8% [24.5–25.1%]) | 32412 (24.9% [24.7–25.2%]) | 20910 (23.7% [23.4–23.9%]) | 33566 (25.7% [25.5–26%]) | 45088 (24.4% [24.2–24.6%]) | 9388 (27.5% [27.1–28%]) |
| Prenatal visits | None | 632 (0.7% [0.7–0.8%]) | 1515 (1.2% [1.1–1.2%]) | 722 (0.8% [0.8–0.9%]) | 1425 (1.1% [1–1.2%]) | 1781 (1% [0.9–1%]) | 366 (1.1% [1–1.2%]) |
|  | 1–6 | 14949 (16.9% [16.6–17.1%]) | 36691 (28.4% [28.1–28.6%]) | 15024 (17.1% [16.8–17.3%]) | 36616 (28.2% [28–28.5%]) | 42847 (23.3% [23.1–23.5%]) | 8793 (25.9% [25.5–26.4%]) |
|  | ≥7 | 72982 (82.4% [82.2–82.7%]) | 91032 (70.4% [70.2–70.7%]) | 72295 (82.1% [81.9–82.4%]) | 91719 (70.7% [70.4–70.9%]) | 139254 (75.7% [75.5–75.9%]) | 24760 (73% [72.5–73.5%]) |
| Maternal education | None | 282 (0.3% [0.3–0.4%]) | 438 (0.3% [0.3–0.4%]) | 365 (0.4% [0.4–0.5%]) | 355 (0.3% [0.2–0.3%]) | 577 (0.3% [0.3–0.3%]) | 143 (0.4% [0.4–0.5%]) |
|  | 1–7 | 11563 (13.1% [12.8–13.3%]) | 16118 (12.5% [12.3–12.6%]) | 12660 (14.4% [14.2–14.6%]) | 15021 (11.6% [11.4–11.7%]) | 23234 (12.6% [12.5–12.8%]) | 4447 (13.1% [12.7–13.5%]) |
|  | 8–11 | 50038 (56.5% [56.2–56.9%]) | 72007 (55.6% [55.4–55.9%]) | 50434 (57.4% [57–57.7%]) | 71611 (55.1% [54.8–55.3%]) | 103291 (56.1% [55.9–56.4%]) | 18754 (55.2% [54.7–55.7%]) |
|  | ≥12 | 26641 (30.1% [29.8–30.4%]) | 40872 (31.6% [31.3–31.8%]) | 24472 (27.8% [27.5–28.1%]) | 43041 (33.1% [32.8–33.4%]) | 56885 (30.9% [30.7–31.1%]) | 10628 (31.3% [30.8–31.8%]) |
| Living with a partner | Living with a partner | 52820 (59.5% [59.2–59.8%]) | 75236 (58% [57.8–58.3%]) | 51237 (58.1% [57.8–58.4%]) | 76819 (59% [58.7–59.3%]) | 108101 (58.6% [58.4–58.9%]) | 19955 (58.6% [58.1–59.1%]) |
|  | Not living with a partner | 35916 (40.5% [40.2–40.8%]) | 54431 (42% [41.7–42.2%]) | 36938 (41.9% [41.6–42.2%]) | 53409 (41% [40.7–41.3%]) | 76256 (41.4% [41.1–41.6%]) | 14091 (41.4% [40.9–41.9%]) |
| Maternal race/skin color | White | 35420 (40.9% [40.5–41.2%]) | 55173 (43.5% [43.2–43.8%]) | 33027 (38.4% [38.1–38.7%]) | 57566 (45.2% [44.9–45.4%]) | 76542 (42.5% [42.2–42.7%]) | 14051 (42.2% [41.7–42.8%]) |
|  | Black | 6199 (7.1% [7–7.3%]) | 9177 (7.2% [7.1–7.4%]) | 6535 (7.6% [7.4–7.8%]) | 8841 (6.9% [6.8–7.1%]) | 12937 (7.2% [7.1–7.3%]) | 2439 (7.3% [7.1–7.6%]) |
|  | Parda (mixed colors) | 44204 (51% [50.7–51.3%]) | 61224 (48.3% [48–48.6%]) | 45546 (52.9% [52.6–53.3%]) | 59882 (47% [46.7–47.3%]) | 88976 (49.4% [49.1–49.6%]) | 16452 (49.5% [48.9–50%]) |
|  | Asian | 375 (0.4% [0.4–0.5%]) | 615 (0.5% [0.4–0.5%]) | 379 (0.4% [0.4–0.5%]) | 611 (0.5% [0.4–0.5%]) | 836 (0.5% [0.4–0.5%]) | 154 (0.5% [0.4–0.5%]) |
|  | Indigenous | 503 (0.6% [0.5–0.6%]) | 589 (0.5% [0.4–0.5%]) | 567 (0.7% [0.6–0.7%]) | 525 (0.4% [0.4–0.4%]) | 922 (0.5% [0.5–0.5%]) | 170 (0.5% [0.4–0.6%]) |
| Region | North | 14348 (8.5% [8.3–8.6%]) | 19320 (7.4% [7.3–7.5%]) | 15252 (8.8% [8.6–8.9%]) | 18416 (7.1% [7.1–7.3%]) | 28532 (7.8% [7.8–7.9%]) | 5136 (7.6% [7.4–7.8%]) |
|  | Northeast | 52121 (30.7% [30.5–31%]) | 69980 (26.7% [26.6–26.9%]) | 53858 (31% [30.8–31.2%]) | 68243 (26.5% [26.3–26.7%]) | 102853 (28.3% [28.1–28.4%]) | 19248 (28.6% [28.3–28.9%]) |
|  | Southeast | 67683 (39.9% [39.7–40.2%]) | 112905 (43.1% [42.9–43.3%]) | 69914 (40.2% [40–40.5%]) | 110674 (43% [42.8–43.2%]) | 152408 (41.9% [41.7–42%]) | 28180 (41.9% [41.5–42.2%]) |
|  | South | 24611 (14.5% [14.3–14.7%]) | 42059 (16.1% [15.9–16.2%]) | 23516 (13.5% [13.4–13.7%]) | 43154 (16.8% [16.6–16.9%]) | 56298 (15.5% [15.3–15.6%]) | 10372 (15.4% [15.1–15.7%]) |
|  | Midwest | 10794 (6.4% [6.3–6.5%]) | 17482 (6.7% [6.6–6.8%]) | 11193 (6.4% [6.3–6.6%]) | 17083 (6.6% [6.5–6.7%]) | 23918 (6.6% [6.5–6.7%]) | 4358 (6.5% [6.3–6.7%]) |
| Newborn race/skin color | White | 68149 (40.4% [40.2–40.7%]) | 113226 (43.6% [43.4–43.8%]) | 66196 (38.3% [38–38.5%]) | 115179 (45.1% [44.9–45.3%]) | 153231 (42.4% [42.2–42.6%]) | 28144 (42.2% [41.8–42.5%]) |
|  | Black | 12046 (7.1% [7–7.3%]) | 18717 (7.2% [7.1–7.3%]) | 13079 (7.6% [7.4–7.7%]) | 17684 (6.9% [6.8–7%]) | 25882 (7.2% [7.1–7.2%]) | 4881 (7.3% [7.1–7.5%]) |
|  | Parda (mixed colors) | 86686 (51.4% [51.2–51.7%]) | 125164 (48.2% [48–48.4%]) | 91789 (53.1% [52.8–53.3%]) | 120061 (47% [46.9–47.2%]) | 178775 (49.5% [49.3–49.6%]) | 33075 (49.5% [49.2–49.9%]) |
|  | Asian | 719 (0.4% [0.4–0.5%]) | 1259 (0.5% [0.5–0.5%]) | 758 (0.4% [0.4–0.5%]) | 1220 (0.5% [0.5–0.5%]) | 1670 (0.5% [0.4–0.5%]) | 308 (0.5% [0.4–0.5%]) |
|  | Indigenous | 983 (0.6% [0.5–0.6%]) | 1214 (0.5% [0.4–0.5%]) | 1145 (0.7% [0.6–0.7%]) | 1052 (0.4% [0.4–0.4%]) | 1851 (0.5% [0.5–0.5%]) | 346 (0.5% [0.5–0.6%]) |
| Sex | Male | 93922 (54.5% [54.3–54.8%]) | 123431 (46.5% [46.3–46.7%]) | 86950 (49.2% [49–49.4%]) | 130403 (50% [49.8–50.2%]) | 183508 (49.7% [49.5–49.9%]) | 33845 (49.6% [49.2–50%]) |
|  | Female | 78335 (45.5% [45.2–45.7%]) | 141797 (53.5% [53.3–53.7%]) | 89741 (50.8% [50.6–51%]) | 130391 (50% [49.8–50.2%]) | 185767 (50.3% [50.1–50.5%]) | 34365 (50.4% [50–50.8%]) |
| Mode of delivery | Vaginal | 15315 (8.9% [8.8–9%]) | 39180 (14.8% [14.6–14.9%]) | 17451 (9.9% [9.7–10%]) | 37044 (14.2% [14.1–14.3%]) | 46988 (12.7% [12.6–12.8%]) | 7507 (11% [10.8–11.2%]) |
|  | Cesarean | 156904 (91.1% [91–91.2%]) | 226001 (85.2% [85.1–85.4%]) | 159195 (90.1% [90–90.3%]) | 223710 (85.8% [85.7–85.9%]) | 322222 (87.3% [87.2–87.4%]) | 60683 (89% [88.8–89.2%]) |
