# Supplementary material for: Maternal characteristics and neonatal outcomes in twin pregnancies in Brazil: A nationwide population-based study, 2015–2024
Source: PLoS One. 2026 Aug 31;21(8):e0355195. doi: 10.1371/journal.pone.0355195 (PMC13529000; doi:10.1371/journal.pone.0355195)
Supplement: Supplementary file 2 [file pone.0355195.s002.docx]

**Manuscript Number:** PONE-D-26-17565

**Review of the article entitled:** Maternal characteristics and neonatal outcomes in twin pregnancies in Brazil: a nationwide population-based study, 2015–2024

Dear Editor,

Initially, I thank you for the opportunity to contribute to the review of this study.

In general, it is a well-written study with great potential to represent the Brazilian reality. The mandatory subheadings are interconnected. However, some clarifications and suggestions need to be addressed. As described below:

**page 97 -** I request that the authors clarify the criterion used to determine the minimum gestational age (24 weeks), since this criterion is not contemplated in the definitions of extreme prematurity or abortion.

**Page 130** - I request clarification from the authors regarding the obtaining of the gestational age variable (the text states: expressed in weeks, obtained based on the best available estimate (last menstruation or clinical evaluation), is this the ultrasound clinical evaluation during pregnancy or the clinical evaluation of the newborn (physical examination at birth)?

**Page 133 -** I request clarification from the authors on how weight control was performed and how data was treated if a diagnosis of fetal centralization occurred?

**Page 136 -** I request details from the authors regarding the statistical tests used. The items are described in a very summarized way. Indicate here in the methodology or in the tables the confounding factors/control of biases used in the tests.

**Page 306 -** I request the authors to include in the study limitations maternal pathologies, such as gestational Diabetes Mellitus and specific hypertensive diseases of pregnancy, and also the evaluation of maternal nutritional status during pregnancy.

Sincerely

Dr Márcia Dornelles Mariot
