# Supplementary material for: Maternal characteristics and neonatal outcomes in twin pregnancies in Brazil: A nationwide population-based study, 2015–2024
Source: PLoS One. 2026 Aug 31;21(8):e0355195. doi: 10.1371/journal.pone.0355195 (PMC13529000; doi:10.1371/journal.pone.0355195)
Supplement: Supplementary file 3 [file pone.0355195.s004.docx]

To Dr. **Elma Izze Da Silva Magalhães**

Academic Editor

PLOS One

Manuscript Number: PONE-D-26-17565

Dear Dr Magalhães,

We appreciate the editor’s and reviewers’ comments on our manuscript entitled **“Maternal characteristics and neonatal outcomes in twin pregnancies in Brazil: a nationwide population-based study, 2015–2024”**.

We are submitting a revised manuscript and a letter responding to the concerns raised by the editor and the reviewers. Changes are highlighted in the manuscript file and addressed in the point-by-point response to the reviewers’ comments. We believe that the changes have satisfactorily addressed their concerns.

The results presented in our study have not been submitted or accepted for publication elsewhere. The manuscript has been read and approved by all authors.

Sincerely,

Dr Fernanda Rebelo

Clinical Research Unit, National Institute of Women’s, Children’s and Adolescents’ Health Fernandes Figueira (IFF), Oswaldo Cruz Foundation (Fiocruz), Rio de Janeiro, RJ, Brazil.Oswaldo Cruz Foundation. Fernandes Figueira Institute. Department of Clinical Research.

E-mail: frebelos@gmail.com

**ANSWERS TO THE REVIEWER’S COMMENTS**

(Authors’ comments are in red and modifications to the original text are highlighted in yellow)

**Academic editor**

Changes required for acceptance:

1. Adequacy of the interpretation of results to the study design: Although the study is correctly presented as descriptive and cross-sectional, some sections of the manuscript use language that suggests independent associations or causal relationships between maternal characteristics and neonatal outcomes. Since no adjusted analytical models or formal association analyses were performed, it is recommended that the wording throughout the manuscript, particularly in the Results and Discussion sections, be revised to replace expressions such as “strongly associated” with descriptions that are consistent with the descriptive nature of the data.

**Response:** We thank the editor and the reviewers for this observation. We reviewed the manuscript, especially the discussion section, to ensure consistency between the study design and the interpretation of the findings. We have also clarified that in the statistical analysis section.

**Changes to the manuscript (Statistical analysis, page 6, lines 141-143):**

Categorical variables were presented as absolute and relative frequencies with corresponding 95% confidence intervals (95% CI). Comparisons between groups and across study years were based on the magnitude of observed differences and their confidence intervals. Because twins share the same pregnancy and, consequently, maternal characteristics, the potential lack of independence between newborns was considered during the analytical strategy. For outcomes defined at the pregnancy level, such as preterm birth and birth weight discordance, each twin pair represents a single biological context, therefore, comparisons using proportions and confidence intervals are appropriate for the purposes of this study.

2. Justify the criterion adopted for the inclusion of pregnancies with a minimum gestational age of 24 weeks;

**Response:** The decision to use 24 weeks as the minimum biologically plausible value was based on two international references (one specifically for twin pregnancies), which were added to the text.

**Changes to the manuscript:**

**New text:**

**(Methods - Study design, population, and eligibility criteria, page 4, lines 96-99)**

Gestational age values below 168 days (24 completed weeks) or above 301 days (43 completed weeks) were excluded according to prespecified eligibility criteria. This range is consistent with procedures adopted by the WHO Technical Advisory Group on Gestational Weight Gain for harmonization of international datasets, in which deliveries outside this interval were flagged for further evaluation [14], and with previous registry-based studies of twin pregnancies [15].

References:
14. World Health Organization. Report on the Fifth Meeting of the WHO Technical Advisory Group on Gestational Weight Gain. Geneva: World Health Organization; 2025.

15. Murray SR, Bhattacharya S, Stock SJ, Pell JP, Norman JE. Gestational age at delivery of twins and perinatal outcomes: a cohort study in Aberdeen, Scotland. Wellcome Open Res. 2019;4:65. doi:10.12688/wellcomeopenres.15211.2.

3. Provide details on the source of the gestational age variable in SINASC, clearly specifying which method was used for its estimation (last menstrual period, obstetric ultrasound, or neonatal clinical assessment);

**Response:** The description of the gestational age variable was expanded to clarify its source and method of ascertainment in SINASC. Specifically, we added that gestational age recorded in SINASC originates from the Declaration of Live Birth and is expressed in completed weeks. According to the instructions for completing the Declaration of Live Birth, when the date of the last menstrual period is unavailable, gestational age may be estimated using other methods, including ultrasonography or clinical examination. We also clarified that the specific method used to determine gestational age for each individual birth is not available in the public SINASC database.

**Changes to the manuscript (Methods - Main variables, page 6, lines 130-135):**

Gestational age, expressed in completed weeks, was obtained from SINASC records and reflects the best estimate available at birth registration. According to the instructions for completing the Declaration of Live Birth, when the date of the last menstrual period is unavailable, gestational age may be estimated using other methods, including ultrasonography or clinical examination. However, the specific method used to determine gestational age for each individual birth is not available in the public SINASC database [12].

12. Brasil. Ministério da Saúde. Secretaria de Vigilância em Saúde. Departamento de Análise Epidemiológica e Vigilância de Doenças Não Transmissíveis. *Declaração de Nascido Vivo: manual de instruções para preenchimento* [Internet]. 4. ed. Brasília: Ministério da Saúde; 2022 [cited 2026 Jun 23]. 80 p. Available from: <http://bvsms.saude.gov.br/bvs/publicacoes/declaracao_nascido_vivo_manual_4ed.pdf>

4. Clarify the procedures used for obtaining and quality control of the birth weight variables;

Response:We clarified that birth weight was obtained from SINASC records as documented in the Declaration of Live Birth and corresponded to the weight recorded at delivery. Records with missing or inconsistent information in key variables, including gestational age, sex, and birth weight, were excluded from the analyses. In addition, as part of the data quality procedures, extreme birth weight values were evaluated using sex- and gestational age-specific z-scores, and records exceeding ±5 standard deviations were excluded to minimize the influence of implausible values on the study estimates. This approach is consistent with previously described methods for identifying implausible birthweight-for-gestational-age combinations in large population datasets and with quality control procedures adopted in studies involving twin populations [1,2].

References:
1. Joseph KS, Kramer MS, Allen AC, Mery LS, Platt RW, Wen SW. Implausible birthweight for gestational age. Am J Epidemiol. 2001;153(2):110–113. doi:10.1093/aje/153.2.110.

2. Beck JJ, Pool R, van de Weijer M, Chen X, Krapohl E, Gordon SD, et al. Genetic meta-analysis of twin birthweight shows high genetic correlation with singleton birthweight. Hum Mol Genet. 2021;30(19):1894–1905. doi:10.1093/hmg/ddab121.

**Changes to the manuscript (Methods - Main variables, page 6, lines 127-128):**

Birth weight was obtained from SINASC records and corresponds to the newborn's weight recorded at delivery in the Declaration of Live Birth.

5. Indicate whether any strategy was adopted to address specific clinical conditions that may influence the interpretation of the findings, such as fetal growth restriction and fetal centralization;

**Response:** As clarified in the revised manuscript, all analyses were based exclusively on information available in SINASC, specifically the birth weight recorded at delivery in the Declaration of Live Birth. The database does not contain information on fetal growth monitoring during pregnancy, estimated fetal weight, fetal growth restriction, Doppler abnormalities, fetal centralization, or other antenatal assessments. Therefore, no specific strategies could be adopted to account for these conditions, and they could not be incorporated into the analyses. These limitations are now explicitly acknowledged in the Discussion.

**Changes to the manuscript (Discussion, pages 18-19, lines 341-343;345-349):**

Despite these strengths, some limitations should be considered. The use of administrative data may be subject to measurement errors, incomplete records, or misclassification of variables, and although several data-cleaning procedures were implemented, including the exclusion of twin pairs with biologically implausible gestational ages, residual inaccuracies cannot be completely ruled out. Interpretation of maternal race/skin color should be undertaken with caution due to known limitations in data quality [33,34]. Furthermore, the database lacks important clinical variables, including chorionicity, use of assisted reproductive technologies, detailed obstetric indications for delivery, maternal nutritional status during pregnancy, information on maternal morbidities such as gestational diabetes and hypertensive disorders of pregnancy, and other relevant maternal or fetal conditions that could influence neonatal outcomes.

6. Expand the description of the statistical analyses, specifying in detail the tests performed and clarifying whether any procedures were used to control for potential confounding factors or biases.

**Response:** We reviewed the statistical analysis section to reflect the descriptive nature of the paper. No procedures to control for potential confounders were adopted. In addition, in the sections “Study design, population, and eligibility criteria” and “Identification of twin pairs” we provide details on how data cleaning and identification of twins was performed and how potential measurement errors and biases were minimized.

**Changes to the manuscript (Methods - Statistical analysis, page 6, lines 141-143;148-150):**

Categorical variables were presented as absolute and relative frequencies with corresponding 95% confidence intervals (95% CI). Comparisons between groups and across study years were based on the magnitude of observed differences and their confidence intervals. Because twins share the same pregnancy and, consequently, maternal characteristics, the potential lack of independence between newborns was considered during the analytical strategy. For outcomes defined at the pregnancy level, such as preterm birth and birth weight discordance, each twin pair represents a single biological context, therefore, comparisons using proportions and confidence intervals are appropriate for the purposes of this study. Temporal variation was assessed through annual frequencies and graphical presentation of the main indicators to identify clinically-relevant changes over time. All analyses were performed using R software, version 4.5.1.

7. The limitations section should be expanded to more explicitly acknowledge the inherent restrictions associated with the use of SINASC.

**Response:** We edited the Strengths and Limitations section to recognize the limitations to work with this administrative dataset.

**Changes to the manuscript (Discussion – Strengths and limitations, page 18, lines 341-343;345-349):**

Despite these strengths, some limitations should be considered. The use of administrative data may be subject to measurement errors, incomplete records, or misclassification of variables, and although several data-cleaning procedures were implemented, including the exclusion of twin pairs with biologically implausible gestational ages, residual inaccuracies cannot be completely ruled out. Interpretation of maternal race/skin color should be undertaken with caution due to known limitations in data quality [33,34]. Furthermore, the database lacks important clinical variables, including chorionicity, use of assisted reproductive technologies, detailed obstetric indications for delivery, maternal nutritional status during pregnancy, information on maternal morbidities such as gestational diabetes and hypertensive disorders of pregnancy, and other relevant maternal or fetal conditions that could influence neonatal outcomes.

8. Include the inability to assess relevant clinical information, such as: Gestational diabetes mellitus, hypertensive disorders of pregnancy, maternal nutritional status during pregnancy, chorionicity, fetal presentation, obstetric and fetal complications, and the use of assisted reproductive technologies.

Response: The Limitations section was expanded to more explicitly acknowledge the inherent restrictions associated with the use of SINASC, including the lack of detailed clinical information, the inability to distinguish spontaneous from medically indicated preterm births, and the absence of variables related to pregnancy management and fetal surveillance. These limitations and their implications for the interpretation of the findings are now discussed in greater detail.

**Changes to the manuscript (Discussion – Strengths and limitations, page 18, lines 341-343;345-349):**

Despite these strengths, some limitations should be considered. The use of administrative data may be subject to measurement errors, incomplete records, or misclassification of variables, and although several data-cleaning procedures were implemented, including the exclusion of twin pairs with biologically implausible gestational ages, residual inaccuracies cannot be completely ruled out. Interpretation of maternal race/skin color should be undertaken with caution due to known limitations in data quality [33,34]. Furthermore, the database lacks important clinical variables, including chorionicity, use of assisted reproductive technologies, detailed obstetric indications for delivery, maternal nutritional status during pregnancy, information on maternal morbidities such as gestational diabetes and hypertensive disorders of pregnancy, and other relevant maternal or fetal conditions that could influence neonatal outcomes.

9. Emphasize more clearly that the database does not allow differentiation between spontaneous preterm births and those resulting from medical indication (iatrogenic prematurity). This limitation should be incorporated into the interpretation of the findings and into the discussion of the high frequency of prematurity observed in twin pregnancies.

**Response:** The Discussion section was revised to emphasize that SINASC does not allow for the differentiation between spontaneous preterm births and those resulting from medical indication.

**Changes to the manuscript (Discussion, pages 16, lines 290-295):**

In addition, an important caveat when interpreting the rates of preterm birth observed among twins in this study is the fact that the SINASC does not include data to differentiate between spontaneous and medically indicated preterm births. In the context of twin pregnancies, this distinction is especially important because many births occurring before 37 weeks may occur due to medical indication, and not necessarily because of spontaneous onset of labor [30,31].

**10.1–10.3 Discussion of the high cesarean section rates**

The finding of high cesarean section rates observed in the study is relevant. However, caution is recommended in the interpretation of these results, as SINASC does not provide sufficient clinical information to determine the indications for the procedure. Therefore, the authors are requested to: (10.1) revise any interpretations suggesting an excess of cesarean sections that are not directly supported by the available data; (10.2) highlight that it is not possible to distinguish clinically indicated cesarean sections from potentially avoidable procedures; and (10.3) expand the discussion by comparing the observed findings with international recommendations and evidence regarding the management of twin pregnancies.

**Response:** We revised the discussion to address these concerns. We clarified that SINASC does not provide information on fetal presentation, chorionicity, obstetric indications, maternal or fetal complications, or the use of assisted reproductive technologies. Consequently, it is not possible to determine whether the observed cesarean deliveries were clinically indicated or potentially avoidable. We also revised the wording throughout the Discussion to ensure that the findings are presented descriptively and without implying inappropriate or excessive use of cesarean delivery.

**Changes to the manuscript (Discussion, page 17, lines 310-318):**

Cesarean delivery was the predominant mode of birth, accounting for more than 87% of deliveries. This finding is consistent with previous reports showing high cesarean rates in twin pregnancies both in Brazil and internationally [6–8,13,30]. The persistently high frequency observed across all regions is also consistent with the broader obstetric context of Brazil, a country that has historically reported among the highest cesarean rates worldwide [26,29]. However, its interpretation requires caution, as SINASC does not provide information on fetal presentation, chorionicity, obstetric indications, maternal or fetal complications, or the use of assisted reproductive technologies. Thus, it is not possible to determine whether these cesarean deliveries were clinically indicated or potentially avoidable.

11. Including formal temporal trend analyses would substantially strengthen the manuscript, allowing assessment of whether the variations observed between 2015 and 2024 represent statistically significant changes over time. Although such analyses are not essential to address the primary objective of the study, the authors are strongly encouraged to consider their inclusion or, alternatively, justify why they were not performed.

**Response:** Although formal tests for temporal trends could have been performed, we intentionally did not emphasize p-values because the large sample size would almost certainly yield statistically significant results for even negligible temporal changes. Consequently, statistical significance would provide limited additional information beyond what is already evident from the descriptive analyses. Our objective was to evaluate the magnitude and potential clinical relevance of temporal variation rather than whether the observed differences departed from the null hypothesis. We therefore present descriptive estimates and graphical summaries, which provide a more informative assessment of temporal patterns in this context. We revised the Statistical Analysis section to clarify that the temporal variation was assessed through annual frequencies and graphical presentation of the main indicators rather than formal trend testing.

**Changes to the manuscript (Methods - Statistical analysis, pages 6-7, lines 148-150):**

Categorical variables were presented as absolute and relative frequencies with corresponding 95% confidence intervals (95% CI). Comparisons between groups and across study years were based on the magnitude of observed differences and their confidence intervals. Because twins share the same pregnancy and, consequently, maternal characteristics, the potential lack of independence between newborns was considered during the analytical strategy. For outcomes defined at the pregnancy level, such as preterm birth and birth weight discordance, each twin pair represents a single biological context, therefore, comparisons using proportions and confidence intervals are appropriate for the purposes of this study. Temporal variation was assessed through annual frequencies and graphical presentation of the main indicators to identify clinically-relevant changes over time. All analyses were performed using R software, version 4.5.1.

12. The discussion could be strengthened by further addressing several relevant aspects:

12.1 Potential impacts of regional inequalities in healthcare infrastructure, availability of specialized perinatal services, and referral and counter-referral systems;

**Response:** **Response:** The Discussion section was expanded and we incorporated evidence from Brazilian studies [26,27] addressing inequalities in the organization of maternal healthcare services across the country.

References:

26. Leal MC, Szwarcwald CL, Almeida PVB, Aquino EML, Barreto ML, Barros F, et al. Reproductive, maternal, neonatal and child health in the 30 years since the creation of the Unified Health System (SUS). *Cien Saude Colet*. 2018;23(6):1915–1928. doi:10.1590/1413-81232018236.03942018.

27. Albuquerque MV, Viana ALA, Lima LD, Ferreira MP, Fusaro ER, Iozzi FL, et al. Regional inequalities in access to childbirth care in Brazil: geographic accessibility and organization of obstetric services. *Cad Saude Publica*. 2017;33(10):e00087816. doi:10.1590/0102-311X00087816.

**Changes to the manuscript (Discussion, Comparison with the literature, pages 15-16, lines 281-289):**

The high prevalence of preterm birth and low birth weight observed in this study is consistent with previous evidence showing that multiple pregnancies are associated with a substantially higher risk of adverse outcomes compared with singleton pregnancies [6-9]. The regional distribution of these outcomes in Brazil, with higher frequencies in more developed regions, should be interpreted in light of previous national evidence showing a greater concentration of assisted reproductive technologies [25], better access to prenatal and obstetric care [26–28], and more intervention-intensive obstetric care practices [29]. These patterns may also reflect broader regional differences in healthcare infrastructure and service organization. Nationwide studies have documented important inequalities in access to obstetric care across Brazil, including disparities in the availability of intensive care units and specialized maternity services, geographic barriers to childbirth care, and limitations in referral and counter-referral systems within the Brazilian Unified Health System [26,27]. Such structural differences may influence access to timely and appropriate care for high-risk pregnancies, including twin gestations, and should be considered when interpreting regional variations in neonatal outcomes.

12.2 The role of advanced maternal age in the increasing frequency of twin pregnancies and its potential effects on obstetric risk;

We incorporated that aspect to the discussion, bringing a few references [1,32,4,9] indicating that delayed childbearing has contributed to the increasing frequency of twin pregnancies worldwide, both through age-related increases in spontaneous dizygotic twinning and through the greater use of assisted reproductive technologies among older women.

References:

1. Monden CWS, Pison G, Smits J. Twin peaks: more twinning in humans than ever before. Hum Reprod. 2021;36(6):1666–1673.

32.Hall JG. Twinning. Lancet. 2003;362(9385):735-743. doi:10.1016/S0140-6736(03)14237-7.

4.Fauser BCJM, Devroey P, Macklon NS. Multiple birth resulting from ovarian stimulation for subfertility treatment. Lancet. 2005;365(9473):1807–1816.

9. Luke B, Brown MB. Contemporary risks of maternal morbidity and adverse outcomes with increasing maternal age and plurality. Fertil Steril. 2007;88(2):283–293.

**Changes to the manuscript (Discussion, pages 16-17, lines 296-309):**

In our study, women aged ≥ 35 years represented a substantial proportion of mothers of twin pregnancies. Advanced maternal age deserves particular consideration in the interpretation of these findings. Previous studies have shown that delayed childbearing contributes to the increasing frequency of twin pregnancies worldwide [1]. This pattern may reflect both biological and technological mechanisms. The risk of spontaneous dizygotic twinning increases with maternal age, possibly reflecting higher concentrations of follicle-stimulating hormone (FSH) and the resulting increased likelihood of multiple ovulation [32], while older women are also more likely to conceive using assisted reproductive technologies, which further contribute to twinning rates [4]. In addition, maternal age and plurality may jointly contribute to higher frequencies of maternal complications and adverse perinatal outcomes among twin pregnancies [9]. Although our descriptive design does not allow assessment of the independent contribution of maternal age to neonatal outcomes, these findings should be interpreted within the broader demographic and reproductive transitions observed in Brazil and globally.

12.3 Implications of the findings for the planning and organization of maternal and child healthcare services in Brazil.

**Response:**We have revised the end of the discussion section to include this point.

**Changes to the manuscript (Discussion, Conclusions, pages 18-19, lines 351-359):**

The findings of this study have direct implications for the planning and organization of maternal and child healthcare services in Brazil. The high frequency of preterm birth and low birth weight in twin pregnancies reinforces the need to strengthen access to specialized prenatal care, improve referral and counter-referral pathways for high-risk pregnancies, and expand neonatal intensive care capacity. These efforts are particularly relevant in regions with a higher burden of preterm birth and low birth weight, where the demand for specialized obstetric and neonatal services may be greater. At the same time, regional planning should also address inequalities in healthcare infrastructure and access to ensure equitable care for twin pregnancies across the country.

13. Correct the spelling of “áreas” to “areas” (line 267);

Response: The spelling was corrected throughout the revised manuscript.

14. Correct “clinical contexto” to “clinical context” (line 316);

Response: The wording was corrected throughout the revised manuscript.

15. Standardize the terminology used for birth weight throughout the manuscript, adopting a single form consistently (“birth weight” or “birthweight”).

Response: The terminology was standardized throughout the manuscript, and the expression “birth weight” was used consistently in all sections of the revised version.

# **REVIEWER #1**

Reviewer #1: The manuscript “Maternal characteristics and neonatal outcomes in twin pregnancies in Brazil: a nationwide population-based study, 2015–2024” presents a descriptive, population-based study using SINASC data, aiming to describe maternal characteristics and neonatal outcomes in twin pregnancies in Brazil between 2015 and 2024.

Overall, I consider the topic relevant to maternal and child health, especially because twin pregnancies represent a population at higher obstetric and neonatal risk. The use of a national database is a strength of the study, as it allows a broad overview of the Brazilian context and also makes it possible to observe differences across macro-regions. In addition, the final sample is very large, including more than 437,000 live births, corresponding to more than 218,000 complete twin pairs, which gives strength to the descriptive analysis presented.

The manuscript has a clear objective and addresses important outcomes, such as low birth weight, preterm birth, and birth weight discordance between twins. However, I believe that some points still need further clarification, mainly regarding the interpretation of the results and the limitations of the database used.

A first aspect concerns the consistency between the study design and the way some findings are presented. The authors describe the study as descriptive and cross-sectional. However, in some parts of the manuscript, the wording seems to suggest associations between maternal characteristics and neonatal outcomes. One example is the statement that the number of prenatal care visits was the maternal characteristic “most strongly associated” with neonatal outcomes. Since no adjusted models or formal association analyses were presented, I believe it would be more appropriate to use descriptive language, avoiding terms that may suggest causality or independent association. An alternative would be to state that “the largest descriptive difference in the frequency of neonatal outcomes was observed according to the number of prenatal care visits.”

**Response:** We appreciate the reviewer’s comments and reviewed the manuscript, especially the discussion section, to ensure consistency between the study design and the interpretation of the findings (please refer to the changes tracked in the manuscript). We have also clarified the analysis performed in the statistical analysis section.

**Changes to the manuscript (Statistical analysis, page 6, lines 141-143):**

Categorical variables were presented as absolute and relative frequencies with corresponding 95% confidence intervals (95% CI). Comparisons between groups and across study years were based on the magnitude of observed differences and their confidence intervals. Because twins share the same pregnancy and, consequently, maternal characteristics, the potential lack of independence between newborns was considered during the analytical strategy. For outcomes defined at the pregnancy level, such as preterm birth and birth weight discordance, each twin pair represents a single biological context, therefore, comparisons using proportions and confidence intervals are appropriate for the purposes of this study.

Another important point is the interpretation of preterm birth in twin pregnancies. The definition of preterm birth as birth before 37 weeks is appropriate and widely used. Even so, in twin pregnancies, this outcome should be interpreted with caution, since some births before 37 weeks may occur due to medical indication, and not necessarily because of spontaneous onset of labor. Although this issue is mentioned in the discussion, I suggest that the authors make this limitation clearer, emphasizing that SINASC does not allow differentiation between spontaneous and iatrogenic preterm birth.

**Response:** The Discussion section was revised to emphasize that SINASC does not allow for the differentiation between spontaneous preterm births and those resulting from medical indication.

**Changes to the manuscript (Discussion, page 16, lines 290-295):**

In addition, an important caveat when interpreting the rates of preterm birth observed among twins in this study is the fact that the SINASC does not include data to differentiate between spontaneous and medically indicated preterm births. In the context of twin pregnancies, this distinction is especially important because many births occurring before 37 weeks may occur due to medical indication, and not necessarily because of spontaneous onset of labor [30,31].

I also think that the discussion about the high frequency of cesarean delivery should be presented carefully. The finding of approximately 87% cesarean deliveries among twin births is relevant, especially in the Brazilian context. However, the database used does not provide key clinical information, such as fetal presentation, chorionicity, obstetric indication, maternal or fetal complications, or use of assisted reproductive technologies. Therefore, it is not possible to conclude whether these cesarean deliveries were excessive or clinically indicated. I suggest that the authors keep the discussion on the Brazilian context of high cesarean rates, but use more cautious wording, avoiding interpretations that go beyond what the available data can support. I also think that the discussion about the high frequency of cesarean delivery should be presented carefully. The finding of approximately 87% cesarean deliveries among twin births is relevant, especially in the Brazilian context. However, SINASC does not provide enough clinical detail to determine the reasons behind these cesarean deliveries, including whether they were related to fetal presentation, chorionicity, obstetric complications, or other clinical indications. Therefore, it is not possible to conclude whether these cesarean deliveries were excessive or clinically indicated. I suggest that the authors keep the discussion on the Brazilian context of high cesarean rates, but use more cautious wording, avoiding interpretations that go beyond what the available data can support.

**Response:** We revised the discussion section to address these concerns. We acknowledged that the SINASC does not provide information on fetal presentation, chorionicity, obstetric indications, maternal or fetal complications, or the use of assisted reproductive technologies. Consequently, it is not possible to determine whether the observed cesarean deliveries were clinically indicated or potentially avoidable.

**Changes to the manuscript (Discussion, page 17, lines 310-318):**

Cesarean delivery was the predominant mode of birth, accounting for more than 87% of deliveries. This finding is consistent with previous reports showing high cesarean rates in twin pregnancies both in Brazil and internationally [6–8,13,30]. The persistently high frequency observed across all regions is also consistent with the broader obstetric context of Brazil, a country that has historically reported among the highest cesarean rates worldwide [26,29]. However, its interpretation requires caution, as SINASC does not provide information on fetal presentation, chorionicity, obstetric indications, maternal or fetal complications, or the use of assisted reproductive technologies. Thus, it is not possible to determine whether these cesarean deliveries were clinically indicated or potentially avoidable.

In summary, the manuscript addresses an important topic, uses a robust national database, and has the potential to contribute to the literature on twin pregnancies in Brazil.However, I believe that some revisions are needed, especially to better align the interpretation of the results with the descriptive design of the study and to make the limitations related to the use of SINASC clearer. With these adjustments, the study may provide a more consistent contribution to the field of maternal and neonatal health.

**Response:** We appreciate the reviewer’s comments. We reviewed the paper, especially the discussion section, to address the concerns raised by the reviewers and editor.

# **REVIEWER #2**

Reviewer #2: Manuscript Number: PONE-D-26-17565

Review of the article entitled: Maternal characteristics and neonatal outcomes in twin pregnancies in Brazil: a nationwide population-based study, 2015–2024

Some clarifications and suggestions need to be addressed. As described below:

page 97 - I request that the authors clarify the criterion used to determine the minimum gestational age (24 weeks), since this criterion is not contemplated in the definitions of extreme prematurity or abortion.

**Response:** The decision to use 24 weeks as the minimum biologically plausible value was based on two international references (one specifically for twin pregnancies), which were added to the text.

**Changes to the manuscript (Methods - Study design, population, and eligibility criteria, page 4, lines 96-99):**

Gestational age values below 168 days (24 completed weeks) or above 301 days (43 completed weeks) were excluded according to prespecified eligibility criteria. This range is consistent with procedures adopted by the World Health Organization [14], and with previous registry-based studies of twin pregnancies [15].

References:
 14. World Health Organization. Report on the Fifth Meeting of the WHO Technical Advisory Group on Gestational Weight Gain. Geneva: World Health Organization; 2025.

15. Murray SR, Bhattacharya S, Stock SJ, Pell JP, Norman JE. Gestational age at delivery of twins and perinatal outcomes: a cohort study in Aberdeen, Scotland. Wellcome Open Res. 2019;4:65. doi:10.12688/wellcomeopenres.15211.2.

Page 130 - I request clarification from the authors regarding the obtaining of the gestational age variable (the text states: expressed in weeks, obtained based on the best available estimate (last menstruation or clinical evaluation), is this the ultrasound clinical evaluation during pregnancy or the clinical evaluation of the newborn (physical examination at birth)?

**Response:** The description of the gestational age variable was expanded to clarify its source and method of ascertainment in SINASC. Specifically, we added that gestational age recorded in SINASC originates from the Declaration of Live Birth and is expressed in completed weeks. According to the instructions for completing the Declaration of Live Birth, when the date of the last menstrual period is unavailable, gestational age may be estimated using other methods, including ultrasonography or clinical examination. However, the specific method used to determine gestational age for each individual birth is not available in the database.

**Changes to the manuscript (Methods – Main variables, page 6, lines 130-135):**

Gestational age, expressed in completed weeks, was obtained from SINASC records and reflects the best estimate available at birth registration. According to the instructions for completing the Declaration of Live Birth, when the date of the last menstrual period is unavailable, gestational age may be estimated using other methods, including ultrasonography or clinical examination. However, the specific method used to determine gestational age for each individual birth is not available in the public SINASC database [12].

Page 133 - I request clarification from the authors on how weight control was performed and how data was treated if a diagnosis of fetal centralization occurred?

**Response:** We clarified that birth weight was obtained from SINASC records as documented in the Declaration of Live Birth and corresponded to the weight recorded at delivery. Records with missing or inconsistent information in key variables, including gestational age, sex, and birth weight, were excluded from the analyses. In addition, as part of the data quality procedures, extreme birth weight values were evaluated using sex- and gestational age-specific z-scores, and records exceeding ±5 standard deviations were excluded to minimize the influence of implausible values on the study estimates. This approach is consistent with previously described methods for identifying implausible birthweight-for-gestational-age combinations in large population datasets and with quality control procedures adopted in studies involving twin populations [1, 2]. Finally, in the SINASC, there is no information on fetal centralization, so it is not possible to take that into consideration in this analysis.

References:
 1. Joseph KS, Kramer MS, Allen AC, Mery LS, Platt RW, Wen SW. Implausible birthweight for gestational age. Am J Epidemiol. 2001;153(2):110–113. doi:10.1093/aje/153.2.110.

2. Beck JJ, Pool R, van de Weijer M, Chen X, Krapohl E, Gordon SD, et al. Genetic meta-analysis of twin birthweight shows high genetic correlation with singleton birthweight. Hum Mol Genet. 2021;30(19):1894–1905. doi:10.1093/hmg/ddab121.

**Changes to the manuscript (Methods – Main variables, page 6, lines 127-128):**

Birth weight was obtained from SINASC records and corresponds to the newborn's weight recorded at delivery in the Declaration of Live Birth.

Page 136 - I request details from the authors regarding the statistical tests used. The items are described in a very summarized way. Indicate here in the methodology or in the tables the confounding factors/control of biases used in the tests.

**Response:** We reviewed the statistical analysis section to reflect the descriptive nature of the paper. No procedures to control for potential confounders were adopted. In addition, in the sections “Study design, population, and eligibility criteria” and “Identification of twin pairs” we provide details on how data cleaning and identification of twins was performed and how potential measurement errors and biases were minimized.

**Changes to the manuscript (Methods - Statistical analysis, page 6-7, lines 141-143;148-150):**

Categorical variables were presented as absolute and relative frequencies with corresponding 95% confidence intervals (95% CI). Comparisons between groups and across study years were based on the magnitude of observed differences and their confidence intervals. Because twins share the same pregnancy and, consequently, maternal characteristics, the potential lack of independence between newborns was considered during the analytical strategy. For outcomes defined at the pregnancy level, such as preterm birth and birth weight discordance, each twin pair represents a single biological context, therefore, comparisons using proportions and confidence intervals are appropriate for the purposes of this study. Temporal variation was assessed through annual frequencies and graphical presentation of the main indicators to identify clinically-relevant changes over time. All analyses were performed using R software, version 4.5.1.

Page 306 - I request the authors to include in the study limitations maternal pathologies, such as gestational Diabetes Mellitus and specific hypertensive diseases of pregnancy, and also the evaluation of maternal nutritional status during pregnancy.

**Response:** We edited the Strengths and Limitations section to recognize the limitations to work with this administrative dataset.

**Changes to the manuscript (Discussion – Strengths and limitations, page 18, lines 341-343;345-349):**

Despite these strengths, some limitations should be considered. The use of administrative data may be subject to measurement errors, incomplete records, or misclassification of variables, and although several data-cleaning procedures were implemented, including the exclusion of twin pairs with biologically implausible gestational ages, residual inaccuracies cannot be completely ruled out. Interpretation of maternal race/skin color should be undertaken with caution due to known limitations in data quality [33,34]. Furthermore, the database lacks important clinical variables, including chorionicity, use of assisted reproductive technologies, detailed obstetric indications for delivery, maternal nutritional status during pregnancy, information on maternal morbidities such as gestational diabetes and hypertensive disorders of pregnancy, and other relevant maternal or fetal conditions that could influence neonatal outcomes.

**REVIEWER #3**

O manuscrito propõe descrever as características maternas e os desfechos neonatais em gestações gemelares no Brasil entre 2015 e 2024, utilizando dados do Sistema de Informações sobre Nascidos Vivos (SINASC), sendo um estudo populacional de âmbito nacional. Os autores identificaram 218.864 pares de gêmeos completos e examinaram as características sociodemográficas maternas, realização do pré-natal, a via de parto, o baixo peso ao nascer, o parto prematuro e a discordância de peso ao nascer, comparando por região de residência.

O manuscrito está bem escrito, os objetivos são claramente definidos e as conclusões são relevantes para as políticas de saúde materna e neonatal. O mesmo atende aos critérios para publicação na PLOS One, visto que é original, não identifiquei evidências de publicação em outro local, as análises estatísticas estão bem descritas, as conclusões são adequadamente apresentadas e sustentadas pelos dados, o artigo está escrito em inglês padrão (com necessidade de pequenos ajustes listados abaixo), atende às questões éticas em pesquisa e segue as diretrizes de relatórios e disponibilidade de dados.

Diante da avaliação crítica do manuscrito, decidi por aceitar segundo o atendimento às correções listas a seguir:

- Os autores apresentam as frequências anuais dos principais desfechos maternos e neonatais. Embora a abordagem descritiva seja consistente com os objetivos do estudo, a inclusão de análises formais das tendências temporais poderiam fortalecer o estudo, visto que esses resultados auxiliariam a avaliar se as variações observadas ano a ano representam mudanças estatisticamente significativas ao longo do tempo e forneceriam informações adicionais sobre a evolução da gestação gemelar.

**Response:** Although formal tests for temporal trends could have been performed, we intentionally did not emphasize p-values because the large sample size would almost certainly yield statistically significant results for even negligible temporal changes. Consequently, statistical significance would provide limited additional information beyond what is already evident from the descriptive analyses. Our objective was to evaluate the magnitude and potential clinical relevance of temporal variation rather than whether the observed differences departed from the null hypothesis. We therefore present descriptive estimates and graphical summaries, which provide a more informative assessment of temporal patterns in this context. We revised the Statistical Analysis section to clarify that the temporal variation was assessed through annual frequencies and graphical presentation of the main indicators rather than formal trend testing.

**Changes to the manuscript (Methods - Statistical analysis, page 6-7, lines 148-150):**

Categorical variables were presented as absolute and relative frequencies with corresponding 95% confidence intervals (95% CI). Comparisons between groups and across study years were based on the magnitude of observed differences and their confidence intervals. Because twins share the same pregnancy and, consequently, maternal characteristics, the potential lack of independence between newborns was considered during the analytical strategy. For outcomes defined at the pregnancy level, such as preterm birth and birth weight discordance, each twin pair represents a single biological context, therefore, comparisons using proportions and confidence intervals are appropriate for the purposes of this study. Temporal variation was assessed through annual frequencies and graphical presentation of the main indicators to identify clinically-relevant changes over time. All analyses were performed using R software, version 4.5.1.

- A discussão sobre as diferenças regionais poderia ser mais aprofundada. Além dos fatores já mencionados, os autores podem considerar discutir como as disparidades regionais na infraestrutura de saúde, no acesso a serviços perinatais especializados e nos sistemas de encaminhamento podem contribuir para a obstetrícia.

**Response:** The Discussion section was expanded and we incorporated evidence from Brazilian studies [26,27] addressing inequalities in the organization of maternal healthcare services across the country.

References:

26. Leal MC, Szwarcwald CL, Almeida PVB, Aquino EML, Barreto ML, Barros F, et al. Reproductive, maternal, neonatal and child health in the 30 years since the creation of the Unified Health System (SUS). *Cien Saude Colet*. 2018;23(6):1915–1928. doi:10.1590/1413-81232018236.03942018.

27. Albuquerque MV, Viana ALA, Lima LD, Ferreira MP, Fusaro ER, Iozzi FL, et al. Regional inequalities in access to childbirth care in Brazil: geographic accessibility and organization of obstetric services. *Cad Saude Publica*. 2017;33(10):e00087816. doi:10.1590/0102-311X00087816.

**Changes to the manuscript (Discussion, Comparison with the literature, pages 15-16, lines 281-289):**

The high prevalence of preterm birth and low birth weight observed in this study is consistent with previous evidence showing that multiple pregnancies are associated with a substantially higher risk of adverse outcomes compared with singleton pregnancies [6-9]. The regional distribution of these outcomes in Brazil, with higher frequencies in more developed regions, should be interpreted in light of previous national evidence showing a greater concentration of assisted reproductive technologies [25], better access to prenatal and obstetric care [26–28], and more intervention-intensive obstetric care practices [29]. These patterns may also reflect broader regional differences in healthcare infrastructure and service organization. Nationwide studies have documented important inequalities in access to obstetric care across Brazil, including disparities in the availability of intensive care units and specialized maternity services, geographic barriers to childbirth care, and limitations in referral and counter-referral systems within the Brazilian Unified Health System [26,27]. Such structural differences may influence access to timely and appropriate care for high-risk pregnancies, including twin gestations, and should be considered when interpreting regional variations in neonatal outcomes.

- Ainda na discussão, poderia explorar mais o papel da idade materna avançada em gestações gemelares, considerando particularmente as mudanças demográficas e sua potencial relação com o aumento das taxas de gêmeos e do risco obstétrico.

We incorporated that aspect to the discussion, bringing a few references [1,32,4,9] indicating that delayed childbearing has contributed to the increasing frequency of twin pregnancies worldwide, both through age-related increases in spontaneous dizygotic twinning and through the greater use of assisted reproductive technologies among older women.

References:

1. Monden CWS, Pison G, Smits J. Twin peaks: more twinning in humans than ever before. Hum Reprod. 2021;36(6):1666–1673.

32.Hall JG. Twinning. Lancet. 2003;362(9385):735-743. doi:10.1016/S0140-6736(03)14237-7.

4.Fauser BCJM, Devroey P, Macklon NS. Multiple birth resulting from ovarian stimulation for subfertility treatment. Lancet. 2005;365(9473):1807–1816.

9. Luke B, Brown MB. Contemporary risks of maternal morbidity and adverse outcomes with increasing maternal age and plurality. Fertil Steril. 2007;88(2):283–293.

**Changes to the manuscript (Discussion, pages 16-17, lines 296-309):**

In our study, women aged ≥ 35 years represented a substantial proportion of mothers of twin pregnancies. Advanced maternal age deserves particular consideration in the interpretation of these findings. Previous studies have shown that delayed childbearing contributes to the increasing frequency of twin pregnancies worldwide [1]. This pattern may reflect both biological and technological mechanisms. The risk of spontaneous dizygotic twinning increases with maternal age, possibly reflecting higher concentrations of follicle-stimulating hormone (FSH) and the resulting increased likelihood of multiple ovulation [32], while older women are also more likely to conceive using assisted reproductive technologies, which further contribute to twinning rates [4]. In addition, maternal age and plurality may jointly contribute to higher frequencies of maternal complications and adverse perinatal outcomes among twin pregnancies [9]. Although our descriptive design does not allow assessment of the independent contribution of maternal age to neonatal outcomes, these findings should be interpreted within the broader demographic and reproductive transitions observed in Brazil and globally.

- Quanto ao parto cesáreo, os autores reconhecem que as taxas são muito elevadas. A discussão poderia ser ampliada abordando as implicações das taxas extremamente elevadas de cesáreas observadas e como esses resultados se comparam às recomendações internacionais atuais para o manejo de gestações gemelares.

**Response:** We revised the discussion section to address these concerns. We acknowledged that the SINASC does not provide information on fetal presentation, chorionicity, obstetric indications, maternal or fetal complications, or the use of assisted reproductive technologies. Consequently, it is not possible to determine whether the observed cesarean deliveries were clinically indicated or potentially avoidable. The lack of those variables limits any comparisons with national or international recommendations.

**Changes to the manuscript (Discussion, page 17, lines 310-318):**

Cesarean delivery was the predominant mode of birth, accounting for more than 87% of deliveries. This finding is consistent with previous reports showing high cesarean rates in twin pregnancies both in Brazil and internationally [6–8,13,30]. The persistently high frequency observed across all regions is also consistent with the broader obstetric context of Brazil, a country that has historically reported among the highest cesarean rates worldwide [26,29]. However, its interpretation requires caution, as SINASC does not provide information on fetal presentation, chorionicity, obstetric indications, maternal or fetal complications, or the use of assisted reproductive technologies. Thus, it is not possible to determine whether these cesarean deliveries were clinically indicated or potentially avoidable.

- Solicito correção da grafia na palavra “áreas” para “areas” na linha 267; bem como a escrita de “clinical contexto” para “clinical context” na linha 316.

**Response:** The typographical errors identified by the reviewer were corrected throughout the revised manuscript.

- Ao longo do texto, aparecem duas formas diferentes para a escrita do peso ao nascer: “birth weight” e “birthweight”. Sugiro padronizar.

**Response:** The terminology was standardized throughout the manuscript, and the expression “birth weight” was used consistently in all sections of the revised version.

.
