# Supplementary material for: Maternal characteristics and neonatal outcomes in twin pregnancies in Brazil: A nationwide population-based study, 2015–2024
Source: PLoS One. 2026 Aug 31;21(8):e0355195. doi: 10.1371/journal.pone.0355195 (PMC13529000; doi:10.1371/journal.pone.0355195)
Supplement: Peer review history

# Peer review: Decision Letter 0

**DOI:** 10.1371/journal.pone.0355195.r001

**Authors:** Elma Izze Da Silva Magalhães

2 Jun 2026

Dear Dr. Rebelo,

Thank you for submitting your manuscript to PLOS ONE. After careful consideration, we feel that it has merit but does not fully meet PLOS ONE’s publication criteria as it currently stands. Therefore, we invite you to submit a revised version of the manuscript that addresses the points raised during the review process.

==============================

**ACADEMIC EDITOR:**

1. Adequacy of the interpretation of results to the study design: Although the study is correctly presented as descriptive and cross-sectional, some sections of the manuscript use language that suggests independent associations or causal relationships between maternal characteristics and neonatal outcomes. Since no adjusted analytical models or formal association analyses were performed, it is recommended that the wording throughout the manuscript, particularly in the Results and Discussion sections, be revised to replace expressions such as “strongly associated” with descriptions that are consistent with the descriptive nature of the data.

2. Justify the criterion adopted for the inclusion of pregnancies with a minimum gestational age of 24 weeks;

3. Provide details on the source of the gestational age variable in SINASC, clearly specifying which method was used for its estimation (last menstrual period, obstetric ultrasound, or neonatal clinical assessment);

4. Clarify the procedures used for obtaining and quality control of the birth weight variables;

5. Indicate whether any strategy was adopted to address specific clinical conditions that may influence the interpretation of the findings, such as fetal growth restriction and fetal centralization;

6. Expand the description of the statistical analyses, specifying in detail the tests performed and clarifying whether any procedures were used to control for potential confounding factors or biases.

7. The limitations section should be expanded to more explicitly acknowledge the inherent restrictions associated with the use of SINASC.

8. Include the inability to assess relevant clinical information, such as: Gestational diabetes mellitus, hypertensive disorders of pregnancy, maternal nutritional status during pregnancy, chorionicity, fetal presentation, obstetric and fetal complications, and the use of assisted reproductive technologies.

9. Emphasize more clearly that the database does not allow differentiation between spontaneous preterm births and those resulting from medical indication (iatrogenic prematurity). This limitation should be incorporated into the interpretation of the findings and into the discussion of the high frequency of prematurity observed in twin pregnancies.

10. Discussion of the high cesarean section rates: The finding of high cesarean section rates observed in the study is relevant. However, caution is recommended in the interpretation of these results, as SINASC does not provide sufficient clinical information to determine the indications for the procedure. Therefore, the authors are requested to:

10.1 Revise any interpretations suggesting an excess of cesarean sections that are not directly supported by the available data;

10.2 Highlight that it is not possible to distinguish clinically indicated cesarean sections from potentially avoidable procedures;

10.3 Expand the discussion by comparing the observed findings with international recommendations and evidence regarding the management of twin pregnancies.

11. Including formal temporal trend analyses would substantially strengthen the manuscript, allowing assessment of whether the variations observed between 2015 and 2024 represent statistically significant changes over time. Although such analyses are not essential to address the primary objective of the study, the authors are strongly encouraged to consider their inclusion or, alternatively, justify why they were not performed.

12. The discussion could be strengthened by further addressing several relevant aspects:

12.1 Potential impacts of regional inequalities in healthcare infrastructure, availability of specialized perinatal services, and referral and counter-referral systems;

12.2 The role of advanced maternal age in the increasing frequency of twin pregnancies and its potential effects on obstetric risk;

12.3 Implications of the findings for the planning and organization of maternal and child healthcare services in Brazil.

13. Correct the spelling of “áreas” to “areas” (line 267);

14. Correct “clinical contexto” to “clinical context” (line 316);

15. Standardize the terminology used for birth weight throughout the manuscript, adopting a single form consistently (“birth weight” or “birthweight”).

==============================

Please submit your revised manuscript by Jul 17 2026 11:59PM. If you will need more time than this to complete your revisions, please reply to this message or contact the journal office at plosone@plos.org. When you're ready to submit your revision, log on to https://www.editorialmanager.com/pone/ and select the 'Submissions Needing Revision' folder to locate your manuscript file.

- A letter that responds to each point raised by the academic editor and reviewer(s). You should upload this letter as a separate file labeled 'Response to Reviewers'.
- A marked-up copy of your manuscript that highlights changes made to the original version. You should upload this as a separate file labeled 'Revised Manuscript with Track Changes'.
- An unmarked version of your revised paper without tracked changes. You should upload this as a separate file labeled 'Manuscript'.

If you would like to make changes to your financial disclosure, please include your updated statement in your cover letter. Guidelines for resubmitting your figure files are available below the reviewer comments at the end of this letter.

If applicable, we recommend that you deposit your laboratory protocols in protocols.io to enhance the reproducibility of your results. Protocols.io assigns your protocol its own identifier (DOI) so that it can be cited independently in the future. For instructions see: https://journals.plos.org/plosone/s/submission-guidelines#loc-laboratory-protocols. Additionally, PLOS ONE offers an option for publishing peer-reviewed Lab Protocol articles, which describe protocols hosted on protocols.io. Read more information on sharing protocols at https://plos.org/protocols?utm_medium=editorial-email&utm_source=authorletters&utm_campaign=protocols.

As the corresponding author, your ORCID iD is verified in the submission system and will appear in the published article. PLOS supports the use of ORCID, and we encourage all coauthors to register for an ORCID iD and use it as well. Please encourage your coauthors to verify their ORCID iD within the submission system before final acceptance, as unverified ORCID iDs will not appear in the published article. *Only*  the individual author can complete the verification step; PLOS staff *cannot*  verify ORCID iDs on behalf of authors.

We look forward to receiving your revised manuscript.

Kind regards,

Elma Izze Da Silva Magalhães

Academic Editor

PLOS One

Journal Requirements:

When submitting your revision, we need you to address these additional requirements.

1. Please ensure that your manuscript meets PLOS ONE's style requirements, including those for file naming. The PLOS ONE style templates can be found at

https://journals.plos.org/plosone/s/file?id=wjVg/PLOSOne_formatting_sample_main_body.pdf and

https://journals.plos.org/plosone/s/file?id=ba62/PLOSOne_formatting_sample_title_authors_affiliations.pdf.

2. Thank you for stating in your Funding Statement:

“TB received financial support from FAPERJ (Fundação Carlos Chagas Filho de Amparo à Pesquisa do Estado do Rio de Janeiro) through the Doutorado Nota 10 Program (process SEI-260003/016070/2024; https://www.faperj.br). TB also received support from CAPES (Coordenação de Aperfeiçoamento de Pessoal de Nível Superior; https://www.gov.br/capes). The funders had no role in study design, data collection and analysis, decision to publish, or preparation of the manuscript. “

Please provide an amended statement that declares *all* the funding or sources of support (whether external or internal to your organization) received during this study, as detailed online in our guide for authors at http://journals.plos.org/plosone/s/submit-now.  Please also include the statement “There was no additional external funding received for this study.” in your updated Funding Statement.

Please include your amended Funding Statement within your cover letter. We will change the online submission form on your behalf.

3. Thank you for stating the following in the Acknowledgments Section of your manuscript:

“This study was supported by FAPERJ (Fundação Carlos Chagas Filho de Amparo à Pesquisa do Estado do Rio de Janeiro), through the Doutorado Nota 10 Program (process SEI-260003/016070/2024). The authors also acknowledge support from CAPES (Coordenação de Aperfeiçoamento de Pessoal de Nível Superior). The funders had no role in study design, data collection and analysis, decision to publish, or preparation of the manuscript.”

We note that you have provided additional information within the Acknowledgements Section that is not currently declared in your Funding Statement. Please note that funding information should not appear in the Acknowledgments section or other areas of your manuscript. We will only publish funding information present in the Funding Statement section of the online submission form.

Please remove any funding-related text from the manuscript and let us know how you would like to update your Funding Statement. Currently, your Funding Statement reads as follows:

“ TB received financial support from FAPERJ (Fundação Carlos Chagas Filho de Amparo à Pesquisa do Estado do Rio de Janeiro) through the Doutorado Nota 10 Program (process SEI-260003/016070/2024; https://www.faperj.br). TB also received support from CAPES (Coordenação de Aperfeiçoamento de Pessoal de Nível Superior; https://www.gov.br/capes). The funders had no role in study design, data collection and analysis, decision to publish, or preparation of the manuscript. “

Please include your amended statements within your cover letter; we will change the online submission form on your behalf.

4. We note that Figure 5 in your submission contain [map/satellite] images which may be copyrighted. All PLOS content is published under the Creative Commons Attribution License (CC BY 4.0), which means that the manuscript, images, and Supporting Information files will be freely available online, and any third party is permitted to access, download, copy, distribute, and use these materials in any way, even commercially, with proper attribution. For these reasons, we cannot publish previously copyrighted maps or satellite images created using proprietary data, such as Google software (Google Maps, Street View, and Earth). For more information, see our copyright guidelines: http://journals.plos.org/plosone/s/licenses-and-copyright.

We require you to either (1) present written permission from the copyright holder to publish these figures specifically under the CC BY 4.0 license, or (2) remove the figures from your submission:

a. You may seek permission from the original copyright holder of Figure 5 to publish the content specifically under the CC BY 4.0 license.

We recommend that you contact the original copyright holder with the Content Permission Form (http://journals.plos.org/plosone/s/file?id=7c09/content-permission-form.pdf) and the following text:

“I request permission for the open-access journal PLOS ONE to publish XXX under the Creative Commons Attribution License (CCAL) CC BY 4.0 (http://creativecommons.org/licenses/by/4.0/). Please be aware that this license allows unrestricted use and distribution, even commercially, by third parties. Please reply and provide explicit written permission to publish XXX under a CC BY license and complete the attached form.”

Please upload the completed Content Permission Form or other proof of granted permissions as an "Other" file with your submission.

In the figure caption of the copyrighted figure, please include the following text: “Reprinted from [ref] under a CC BY license, with permission from [name of publisher], original copyright [original copyright year].”

b. If you are unable to obtain permission from the original copyright holder to publish these figures under the CC BY 4.0 license or if the copyright holder’s requirements are incompatible with the CC BY 4.0 license, please either i) remove the figure or ii) supply a replacement figure that complies with the CC BY 4.0 license. Please check copyright information on all replacement figures and update the figure caption with source information. If applicable, please specify in the figure caption text when a figure is similar but not identical to the original image and is therefore for illustrative purposes only.

The following resources for replacing copyrighted map figures may be helpful:

USGS National Map Viewer (public domain): http://viewer.nationalmap.gov/viewer/

The Gateway to Astronaut Photography of Earth (public domain): http://eol.jsc.nasa.gov/sseop/clickmap/

Maps at the CIA (public domain): https://www.cia.gov/library/publications/the-world-factbook/index.html and https://www.cia.gov/library/publications/cia-maps-publications/index.html

NASA Earth Observatory (public domain): http://earthobservatory.nasa.gov/

Landsat: http://landsat.visibleearth.nasa.gov/

USGS EROS (Earth Resources Observatory and Science (EROS) Center) (public domain): http://eros.usgs.gov/#

Natural Earth (public domain): http://www.naturalearthdata.com/

5. If the reviewer comments include a recommendation to cite specific previously published works, please review and evaluate these publications to determine whether they are relevant and should be cited. There is no requirement to cite these works unless the editor has indicated otherwise.

6. Please review your reference list to ensure that it is complete and correct. If you have cited papers that have been retracted, please include the rationale for doing so in the manuscript text, or remove these references and replace them with relevant current references. Any changes to the reference list should be mentioned in the rebuttal letter that accompanies your revised manuscript. If you need to cite a retracted article, indicate the article’s retracted status in the References list and also include a citation and full reference for the retraction notice.

Reviewers' comments:

Reviewer's Responses to Questions

**Comments to the Author**

1. Is the manuscript technically sound, and do the data support the conclusions?

Reviewer #1: Partly

Reviewer #2: Yes

Reviewer #3: Yes

**********

2. Has the statistical analysis been performed appropriately and rigorously?

Reviewer #1: Yes

Reviewer #2: N/A

Reviewer #3: Yes

**********

3. Have the authors made all data underlying the findings in their manuscript fully available?

The PLOS Data policy

Reviewer #1: Yes

Reviewer #2: Yes

Reviewer #3: Yes

**********

4. Is the manuscript presented in an intelligible fashion and written in standard English?

Reviewer #1: Yes

Reviewer #2: Yes

Reviewer #3: Yes

**********

Reviewer #1: The manuscript “Maternal characteristics and neonatal outcomes in twin pregnancies in Brazil: a nationwide population-based study, 2015–2024” presents a descriptive, population-based study using SINASC data, aiming to describe maternal characteristics and neonatal outcomes in twin pregnancies in Brazil between 2015 and 2024.

Overall, I consider the topic relevant to maternal and child health, especially because twin pregnancies represent a population at higher obstetric and neonatal risk. The use of a national database is a strength of the study, as it allows a broad overview of the Brazilian context and also makes it possible to observe differences across macro-regions. In addition, the final sample is very large, including more than 437,000 live births, corresponding to more than 218,000 complete twin pairs, which gives strength to the descriptive analysis presented.

The manuscript has a clear objective and addresses important outcomes, such as low birth weight, preterm birth, and birth weight discordance between twins. However, I believe that some points still need further clarification, mainly regarding the interpretation of the results and the limitations of the database used.

A first aspect concerns the consistency between the study design and the way some findings are presented. The authors describe the study as descriptive and cross-sectional. However, in some parts of the manuscript, the wording seems to suggest associations between maternal characteristics and neonatal outcomes. One example is the statement that the number of prenatal care visits was the maternal characteristic “most strongly associated” with neonatal outcomes. Since no adjusted models or formal association analyses were presented, I believe it would be more appropriate to use descriptive language, avoiding terms that may suggest causality or independent association. An alternative would be to state that “the largest descriptive difference in the frequency of neonatal outcomes was observed according to the number of prenatal care visits.”

Another important point is the interpretation of preterm birth in twin pregnancies. The definition of preterm birth as birth before 37 weeks is appropriate and widely used. Even so, in twin pregnancies, this outcome should be interpreted with caution, since some births before 37 weeks may occur due to medical indication, and not necessarily because of spontaneous onset of labor. Although this issue is mentioned in the discussion, I suggest that the authors make this limitation clearer, emphasizing that SINASC does not allow differentiation between spontaneous and iatrogenic preterm birth.

I also think that the discussion about the high frequency of cesarean delivery should be presented carefully. The finding of approximately 87% cesarean deliveries among twin births is relevant, especially in the Brazilian context. However, the database used does not provide key clinical information, such as fetal presentation, chorionicity, obstetric indication, maternal or fetal complications, or use of assisted reproductive technologies. Therefore, it is not possible to conclude whether these cesarean deliveries were excessive or clinically indicated. I suggest that the authors keep the discussion on the Brazilian context of high cesarean rates, but use more cautious wording, avoiding interpretations that go beyond what the available data can support. I also think that the discussion about the high frequency of cesarean delivery should be presented carefully. The finding of approximately 87% cesarean deliveries among twin births is relevant, especially in the Brazilian context. However, SINASC does not provide enough clinical detail to determine the reasons behind these cesarean deliveries, including whether they were related to fetal presentation, chorionicity, obstetric complications, or other clinical indications. Therefore, it is not possible to conclude whether these cesarean deliveries were excessive or clinically indicated. I suggest that the authors keep the discussion on the Brazilian context of high cesarean rates, but use more cautious wording, avoiding interpretations that go beyond what the available data can support.

In summary, the manuscript addresses an important topic, uses a robust national database, and has the potential to contribute to the literature on twin pregnancies in Brazil.However, I believe that some revisions are needed, especially to better align the interpretation of the results with the descriptive design of the study and to make the limitations related to the use of SINASC clearer. With these adjustments, the study may provide a more consistent contribution to the field of maternal and neonatal health.

Reviewer #2: Manuscript Number: PONE-D-26-17565

Review of the article entitled: Maternal characteristics and neonatal outcomes in twin pregnancies in Brazil: a nationwide population-based study, 2015–2024

Some clarifications and suggestions need to be addressed. As described below:

page 97 - I request that the authors clarify the criterion used to determine the minimum gestational age (24 weeks), since this criterion is not contemplated in the definitions of extreme prematurity or abortion.

Page 130 - I request clarification from the authors regarding the obtaining of the gestational age variable (the text states: expressed in weeks, obtained based on the best available estimate (last menstruation or clinical evaluation), is this the ultrasound clinical evaluation during pregnancy or the clinical evaluation of the newborn (physical examination at birth)?

Page 133 - I request clarification from the authors on how weight control was performed and how data was treated if a diagnosis of fetal centralization occurred?

Page 136 - I request details from the authors regarding the statistical tests used. The items are described in a very summarized way. Indicate here in the methodology or in the tables the confounding factors/control of biases used in the tests.

Page 306 - I request the authors to include in the study limitations maternal pathologies, such as gestational Diabetes Mellitus and specific hypertensive diseases of pregnancy, and also the evaluation of maternal nutritional status during pregnancy.

Sincerely

Reviewer #3: O manuscrito propõe descrever as características maternas e os desfechos neonatais em gestações gemelares no Brasil entre 2015 e 2024, utilizando dados do Sistema de Informações sobre Nascidos Vivos (SINASC), sendo um estudo populacional de âmbito nacional. Os autores identificaram 218.864 pares de gêmeos completos e examinaram as características sociodemográficas maternas, realização do pré-natal, a via de parto, o baixo peso ao nascer, o parto prematuro e a discordância de peso ao nascer, comparando por região de residência.

O manuscrito está bem escrito, os objetivos são claramente definidos e as conclusões são relevantes para as políticas de saúde materna e neonatal. O mesmo atende aos critérios para publicação na PLOS One, visto que é original, não identifiquei evidências de publicação em outro local, as análises estatísticas estão bem descritas, as conclusões são adequadamente apresentadas e sustentadas pelos dados, o artigo está escrito em inglês padrão (com necessidade de pequenos ajustes listados abaixo), atende às questões éticas em pesquisa e segue as diretrizes de relatórios e disponibilidade de dados.

Diante da avaliação crítica do manuscrito, decidi por aceitar segundo o atendimento às correções listas a seguir:

- Os autores apresentam as frequências anuais dos principais desfechos maternos e neonatais. Embora a abordagem descritiva seja consistente com os objetivos do estudo, a inclusão de análises formais das tendências temporais poderiam fortalecer o estudo, visto que esses resultados auxiliariam a avaliar se as variações observadas ano a ano representam mudanças estatisticamente significativas ao longo do tempo e forneceriam informações adicionais sobre a evolução da gestação gemelar.

- A discussão sobre as diferenças regionais poderia ser mais aprofundada. Além dos fatores já mencionados, os autores podem considerar discutir como as disparidades regionais na infraestrutura de saúde, no acesso a serviços perinatais especializados e nos sistemas de encaminhamento podem contribuir para a obstetrícia.

- Ainda na discussão, poderia explorar mais o papel da idade materna avançada em gestações gemelares, considerando particularmente as mudanças demográficas e sua potencial relação com o aumento das taxas de gêmeos e do risco obstétrico.

- Quanto ao parto cesáreo, os autores reconhecem que as taxas são muito elevadas. A discussão poderia ser ampliada abordando as implicações das taxas extremamente elevadas de cesáreas observadas e como esses resultados se comparam às recomendações internacionais atuais para o manejo de gestações gemelares.

- Solicito correção da grafia na palavra “áreas” para “areas” na linha 267; bem como a escrita de “clinical contexto” para “clinical context” na linha 316.

- Ao longo do texto, aparecem duas formas diferentes para a escrita do peso ao nascer: “birth weight” e “birthweight”. Sugiro padronizar.

**********

what does this mean?). If published, this will include your full peer review and any attached files.

If you choose “no”, your identity will remain anonymous but your review may still be made public.

**Do you want your identity to be public for this peer review?**  For information about this choice, including consent withdrawal, please see our Privacy Policy

Reviewer #1: No

Reviewer #2: No

Reviewer #3: No

**********

[NOTE: If reviewer comments were submitted as an attachment file, they will be attached to this email and accessible via the submission site. Please log into your account, locate the manuscript record, and check for the action link "View Attachments". If this link does not appear, there are no attachment files.]

To ensure your figures meet our technical requirements, please review our figure guidelines: https://journals.plos.org/plosone/s/figures

You may also use PLOS’s free figure tool, NAAS, to help you prepare publication quality figures: https://journals.plos.org/plosone/s/figures#loc-tools-for-figure-preparation.

NAAS will assess whether your figures meet our technical requirements by comparing each figure against our figure specifications.

Attachment Submitted filename: plos one review.docx

# Author comment: Author response to Decision Letter 1

**DOI:** 10.1371/journal.pone.0355195.r002

15 Jul 2026

ANSWERS TO THE REVIEWER’S COMMENTS

(Authors’ comments are in red and modifications to the original text are highlighted in yellow)

Academic editor

Changes required for acceptance:

1. Adequacy of the interpretation of results to the study design: Although the study is correctly presented as descriptive and cross-sectional, some sections of the manuscript use language that suggests independent associations or causal relationships between maternal characteristics and neonatal outcomes. Since no adjusted analytical models or formal association analyses were performed, it is recommended that the wording throughout the manuscript, particularly in the Results and Discussion sections, be revised to replace expressions such as “strongly associated” with descriptions that are consistent with the descriptive nature of the data.

Response: We thank the editor and the reviewers for this observation. We reviewed the manuscript, especially the discussion section, to ensure consistency between the study design and the interpretation of the findings. We have also clarified that in the statistical analysis section.

Changes to the manuscript (Statistical analysis, page 6, lines 141-143):

Categorical variables were presented as absolute and relative frequencies with corresponding 95% confidence intervals (95% CI). Comparisons between groups and across study years were based on the magnitude of observed differences and their confidence intervals. Because twins share the same pregnancy and, consequently, maternal characteristics, the potential lack of independence between newborns was considered during the analytical strategy. For outcomes defined at the pregnancy level, such as preterm birth and birth weight discordance, each twin pair represents a single biological context, therefore, comparisons using proportions and confidence intervals are appropriate for the purposes of this study.

2. Justify the criterion adopted for the inclusion of pregnancies with a minimum gestational age of 24 weeks;

Response: The decision to use 24 weeks as the minimum biologically plausible value was based on two international references (one specifically for twin pregnancies), which were added to the text.

Changes to the manuscript:

New text:

(Methods - Study design, population, and eligibility criteria, page 4, lines 96-99)

Gestational age values below 168 days (24 completed weeks) or above 301 days (43 completed weeks) were excluded according to prespecified eligibility criteria. This range is consistent with procedures adopted by the WHO Technical Advisory Group on Gestational Weight Gain for harmonization of international datasets, in which deliveries outside this interval were flagged for further evaluation [14], and with previous registry-based studies of twin pregnancies [15].

References:

14. World Health Organization. Report on the Fifth Meeting of the WHO Technical Advisory Group on Gestational Weight Gain. Geneva: World Health Organization; 2025.

15. Murray SR, Bhattacharya S, Stock SJ, Pell JP, Norman JE. Gestational age at delivery of twins and perinatal outcomes: a cohort study in Aberdeen, Scotland. Wellcome Open Res. 2019;4:65. doi:10.12688/wellcomeopenres.15211.2.

3. Provide details on the source of the gestational age variable in SINASC, clearly specifying which method was used for its estimation (last menstrual period, obstetric ultrasound, or neonatal clinical assessment);

Response: The description of the gestational age variable was expanded to clarify its source and method of ascertainment in SINASC. Specifically, we added that gestational age recorded in SINASC originates from the Declaration of Live Birth and is expressed in completed weeks. According to the instructions for completing the Declaration of Live Birth, when the date of the last menstrual period is unavailable, gestational age may be estimated using other methods, including ultrasonography or clinical examination. We also clarified that the specific method used to determine gestational age for each individual birth is not available in the public SINASC database.

Changes to the manuscript (Methods - Main variables, page 6, lines 130-135):

Gestational age, expressed in completed weeks, was obtained from SINASC records and reflects the best estimate available at birth registration. According to the instructions for completing the Declaration of Live Birth, when the date of the last menstrual period is unavailable, gestational age may be estimated using other methods, including ultrasonography or clinical examination. However, the specific method used to determine gestational age for each individual birth is not available in the public SINASC database [12].

12. Brasil. Ministério da Saúde. Secretaria de Vigilância em Saúde. Departamento de Análise Epidemiológica e Vigilância de Doenças Não Transmissíveis. Declaração de Nascido Vivo: manual de instruções para preenchimento [Internet]. 4. ed. Brasília: Ministério da Saúde; 2022 [cited 2026 Jun 23]. 80 p. Available from: http://bvsms.saude.gov.br/bvs/publicacoes/declaracao_nascido_vivo_manual_4ed.pdf

4. Clarify the procedures used for obtaining and quality control of the birth weight variables;

Response:We clarified that birth weight was obtained from SINASC records as documented in the Declaration of Live Birth and corresponded to the weight recorded at delivery. Records with missing or inconsistent information in key variables, including gestational age, sex, and birth weight, were excluded from the analyses. In addition, as part of the data quality procedures, extreme birth weight values were evaluated using sex- and gestational age-specific z-scores, and records exceeding ±5 standard deviations were excluded to minimize the influence of implausible values on the study estimates. This approach is consistent with previously described methods for identifying implausible birthweight-for-gestational-age combinations in large population datasets and with quality control procedures adopted in studies involving twin populations [1,2].

References:

1. Joseph KS, Kramer MS, Allen AC, Mery LS, Platt RW, Wen SW. Implausible birthweight for gestational age. Am J Epidemiol. 2001;153(2):110–113. doi:10.1093/aje/153.2.110.

2. Beck JJ, Pool R, van de Weijer M, Chen X, Krapohl E, Gordon SD, et al. Genetic meta-analysis of twin birthweight shows high genetic correlation with singleton birthweight. Hum Mol Genet. 2021;30(19):1894–1905. doi:10.1093/hmg/ddab121.

Changes to the manuscript (Methods - Main variables, page 6, lines 127-128):

Birth weight was obtained from SINASC records and corresponds to the newborn's weight recorded at delivery in the Declaration of Live Birth.

5. Indicate whether any strategy was adopted to address specific clinical conditions that may influence the interpretation of the findings, such as fetal growth restriction and fetal centralization;

Response: As clarified in the revised manuscript, all analyses were based exclusively on information available in SINASC, specifically the birth weight recorded at delivery in the Declaration of Live Birth. The database does not contain information on fetal growth monitoring during pregnancy, estimated fetal weight, fetal growth restriction, Doppler abnormalities, fetal centralization, or other antenatal assessments. Therefore, no specific strategies could be adopted to account for these conditions, and they could not be incorporated into the analyses. These limitations are now explicitly acknowledged in the Discussion.

Changes to the manuscript (Discussion, pages 18-19, lines 341-343;345-349):

Despite these strengths, some limitations should be considered. The use of administrative data may be subject to measurement errors, incomplete records, or misclassification of variables, and although several data-cleaning procedures were implemented, including the exclusion of twin pairs with biologically implausible gestational ages, residual inaccuracies cannot be completely ruled out. Interpretation of maternal race/skin color should be undertaken with caution due to known limitations in data quality [33,34]. Furthermore, the database lacks important clinical variables, including chorionicity, use of assisted reproductive technologies, detailed obstetric indications for delivery, maternal nutritional status during pregnancy, information on maternal morbidities such as gestational diabetes and hypertensive disorders of pregnancy, and other relevant maternal or fetal conditions that could influence neonatal outcomes.

6. Expand the description of the statistical analyses, specifying in detail the tests performed and clarifying whether any procedures were used to control for potential confounding factors or biases.

Response: We reviewed the statistical analysis section to reflect the descriptive nature of the paper. No procedures to control for potential confounders were adopted. In addition, in the sections “Study design, population, and eligibility criteria” and “Identification of twin pairs” we provide details on how data cleaning and identification of twins was performed and how potential measurement errors and biases were minimized.

Changes to the manuscript (Methods - Statistical analysis, page 6, lines 141-143;148-150):

Categorical variables were presented as absolute and relative frequencies with corresponding 95% confidence intervals (95% CI). Comparisons between groups and across study years were based on the magnitude of observed differences and their confidence intervals. Because twins share the same pregnancy and, consequently, maternal characteristics, the potential lack of independence between newborns was considered during the analytical strategy. For outcomes defined at the pregnancy level, such as preterm birth and birth weight discordance, each twin pair represents a single biological context, therefore, comparisons using proportions and confidence intervals are appropriate for the purposes of this study. Temporal variation was assessed through annual frequencies and graphical presentation of the main indicators to identify clinically-relevant changes over time. All analyses were performed using R software, version 4.5.1.

7. The limitations section should be expanded to more explicitly acknowledge the inherent restrictions associated with the use of SINASC.

Response: We edited the Strengths and Limitations section to recognize the limitations to work with this administrative dataset.

Changes to the manuscript (Discussion – Strengths and limitations, page 18, lines 341-343;345-349):

Despite these strengths, some limitations should be considered. The use of administrative data may be subject to measurement errors, incomplete records, or misclassification of variables, and although several data-cleaning procedures were implemented, including the exclusion of twin pairs with biologically implausible gestational ages, residual inaccuracies cannot be completely ruled out. Interpretation of maternal race/skin color should be undertaken with caution due to known limitations in data quality [33,34]. Furthermore, the database lacks important clinical variables, including chorionicity, use of assisted reproductive technologies, detailed obstetric indications for delivery, maternal nutritional status during pregnancy, information on maternal morbidities such as gestational diabetes and hypertensive disorders of pregnancy, and other relevant maternal or fetal conditions that could influence neonatal outcomes.

8. Include the inability to assess relevant clinical information, such as: Gestational diabetes mellitus, hypertensive disorders of pregnancy, maternal nutritional status during pregnancy, chorionicity, fetal presentation, obstetric and fetal complications, and the use of assisted reproductive technologies.

Response: The Limitations section was expanded to more explicitly acknowledge the inherent restrictions associated with the use of SINASC, including the lack of detailed clinical information, the inability to distinguish spontaneous from medically indicated preterm births, and the absence of variables related to pregnancy management and fetal surveillance. These limitations and their implications for the interpretation of the findings are now discussed in greater detail.

Changes to the manuscript (Discussion – Strengths and limitations, page 18, lines 341-343;345-349):

Despite these strengths, some limitations should be considered. The use of administrative data may be subject to measurement errors, incomplete records, or misclassification of variables, and although several data-cleaning procedures were implemented, including the exclusion of twin pairs with biologically implausible gestational ages, residual inaccuracies cannot be completely ruled out. Interpretation of maternal race/skin color should be undertaken with caution due to known limitations in data quality [33,34]. Furthermore, the database lacks important clinical variables, including chorionicity, use of assisted reproductive technologies, detailed obstetric indications for delivery, maternal nutritional status during pregnancy, information on maternal morbidities such as gestational diabetes and hypertensive disorders of pregnancy, and other relevant maternal or fetal conditions that could influence neonatal outcomes.

9. Emphasize more clearly that the database does not allow differentiation between spontaneous preterm births and those resulting from medical indication (iatrogenic prematurity). This limitation should be incorporated into the interpretation of the findings and into the discussion of the high frequency of prematurity observed in twin pregnancies.

Response: The Discussion section was revised to emphasize that SINASC does not allow for the differentiation between spontaneous preterm births and those resulting from medical indication.

Changes to the manuscript (Discussion, pages 16, lines 290-295):

In addition, an important caveat when interpreting the rates of preterm birth observed among twins in this study is the fact that the SINASC does not include data to differentiate between spontaneous and medically indicated preterm births. In the context of twin pregnancies, this distinction is especially important because many births occurring before 37 weeks may occur due to medical indication, and not necessarily because of spontaneous onset of labor [30,31].

10.1–10.3 Discussion of the high cesarean section rates

The finding of high cesarean section rates observed in the study is relevant. However, caution is recommended in the interpretation of these results, as SINASC does not provide sufficient clinical information to determine the indications for the procedure. Therefore, the authors are requested to: (10.1) revise any interpretations suggesting an excess of cesarean sections that are not directly supported by the available data; (10.2) highlight that it is not possible to distinguish clinically indicated cesarean sections from potentially avoidable procedures; and (10.3) expand the discussion by comparing the observed findings with international recommendations and evidence regarding the management of twin pregnancies.

Response: We revised the discussion to address these concerns. We clarified that SINASC does not provide information on fetal presentation, chorionicity, obstetric indications, maternal or fetal complications, or the use of assisted reproductive technologies. Consequently, it is not possible to determine whether the observed cesarean deliveries were clinically indicated or potentially avoidable. We also revised the wording throughout the Discussion to ensure that the findings are presented descriptively and without implying inappropriate or excessive use of cesarean delivery.

Changes to the manuscript (Discussion, page 17, lines 310-318):

Cesarean delivery was the predominant mode of birth, accounting for more than 87% of deliveries. This finding is consistent with previous reports showing high cesarean rates in twin pregnancies both in Brazil and internationally [6–8,13,30]. The persistently high frequency observed across all regions is also consistent with the broader obstetric context of Brazil, a country that has historically reported among the highest cesarean rates worldwide [26,29]. However, its interpretation

Attachment Submitted filename: Response to Reviewers.docx

# Editor report: Decision Letter 1

**DOI:** 10.1371/journal.pone.0355195.r003

**Authors:** Elma Izze Da Silva Magalhães

20 Jul 2026

Maternal characteristics and neonatal outcomes in twin pregnancies in Brazil: a nationwide population-based study, 2015–2024

PONE-D-26-17565R1

Dear Dr. Rebelo,

We’re pleased to inform you that your manuscript has been judged scientifically suitable for publication and will be formally accepted for publication once it meets all outstanding technical requirements.

Within one week, you’ll receive an e-mail detailing the required amendments. When these have been addressed, you’ll receive a formal acceptance letter and your manuscript will be scheduled for publication.

An invoice will be generated when your article is formally accepted. Please note, if your institution has a publishing partnership with PLOS and your article meets the relevant criteria, all or part of your publication costs will be covered. Please make sure your user information is up-to-date by logging into Editorial Manager at Editorial Manager® and clicking the ‘Update My Information' link at the top of the page. For questions related to billing, please contact billing support.

If your institution or institutions have a press office, please notify them about your upcoming paper to help maximize its impact. If they’ll be preparing press materials, please inform our press team as soon as possible -- no later than 48 hours after receiving the formal acceptance. Your manuscript will remain under strict press embargo until 2 pm Eastern Time on the date of publication. For more information, please contact onepress@plos.org.

Kind regards,

Elma Izze Da Silva Magalhães

Academic Editor

PLOS One

# Editor report: Acceptance letter

**DOI:** 10.1371/journal.pone.0355195.r004

**Authors:** Elma Izze Da Silva Magalhães

PONE-D-26-17565R1

PLOS One

Dear Dr. Rebelo,

I'm pleased to inform you that your manuscript has been deemed suitable for publication in PLOS One. Congratulations! Your manuscript is now being handed over to our production team.

At this stage, our production department will prepare your paper for publication. This includes ensuring the following:

* All references, tables, and figures are properly cited

* All relevant supporting information is included in the manuscript submission,

* There are no issues that prevent the paper from being properly typeset

You will receive further instructions from the production team, including instructions on how to review your proof when it is ready. Please keep in mind that we are working through a large volume of accepted articles, so please give us a few days to review your paper and let you know the next and final steps.

Lastly, if your institution or institutions have a press office, please let them know about your upcoming paper now to help maximize its impact. If they'll be preparing press materials, please inform our press team within the next 48 hours. Your manuscript will remain under strict press embargo until 2 pm Eastern Time on the date of publication. For more information, please contact onepress@plos.org.

You will receive an invoice from PLOS for your publication fee after your manuscript has reached the completed accept phase. If you receive an email requesting payment before acceptance or for any other service, this may be a phishing scheme. Learn how to identify phishing emails and protect your accounts at https://explore.plos.org/phishing.

If we can help with anything else, please email us at customercare@plos.org.

Thank you for submitting your work to PLOS One and supporting open access.

Kind regards,

PLOS ONE Editorial Office Staff

on behalf of

Dr. Elma Izze Da Silva Magalhães

Academic Editor

PLOS One
